# Rigid Cooperation of Per1 and Per2 proteins

**DOI:** 10.1038/srep32769

**Published:** 2016-09-09

**Authors:** Hiroyuki Tamiya, Sumito Ogawa, Yasuyoshi Ouchi, Masahiro Akishita

**Affiliations:** 1 Department of Geriatric Medicine, Graduate School of Medicine, The University of Tokyo, Tokyo, Japan; 2 Federation of National Public Service Personnel Mutual Aid Associations Toranomon Hospital, Tokyo, Japan

## Abstract

Period circadian clock (Per) genes *Per1* and *Per2* have essential roles in circadian oscillation. In this study, we identified a new role of *Per1*-*Per2* cooperation, and its mechanism, using our new experimental methods. Under constant light conditions, the period length of *Per1* and *Per2* knockout mice depended on the copy number ratio of *Per1:Per2*. We then established a light-emitting diode-based lighting system that can generate any pattern of light intensity. Under gradually changing light in the absence of phase shift with different periods, both *Per1*^(−/−)^ and *Per2*^(−/−)^ mice were entrained to a broader range of period length than wild-type mice. To analyse *Per1*-*Per2* cooperative roles at the cell culture level, we established a *Per2* knockout-rescue system, which can detect period shortening in a familial advanced sleep phase syndrome (FASPS) mutant. Upon introduction of the *Per1* coding region in this system, we saw period shortening. In conclusion, short period-associated protein Per1 and long period-associated Per2 cooperated to rigidly confine the circadian period to “circa” 24-h. These results suggest that the rigid circadian rhythm maintained through the cooperation of Per1-Per2 could negatively impact modern society, in which the use of artificial lighting is ubiquitous, and result in circadian disorders, including delirium.

“Circadian” comes from the Latin *circa* (about) and *dies* (day). Many physiological functions have diurnal variation^1^,^2^,^3^ that follows an approximately 24-h cycle and is regulated by an endogenous circadian clock^4^; dysregulation of these functions leads to circadian disorders, including delirium^5^,^6^. The mechanism underlying the mammalian circadian clock is a transcriptional network that consists of a feedback loop of about 20 genes (clock genes)^7^,^8^. Because every cell in the body has a circadian clock, we can see circadian oscillation in fibroblasts transfected with plasmids encoding luciferase under the control of a circadian clock gene promoter and cultured in luciferin-containing medium^9^. To coordinate the clock in many cells in mammals, the suprachiasmatic nucleus (SCN) in the hypothalamus functions as a central clock^10^,^11^. The SCN entrains clocks of cells in individual organs (peripheral clocks) through neural and humoral mechanisms. The SCN is also important because SCN neurons have an intercellular coupling (synchronization) mechanism that makes the circadian oscillation robust and self-sustainable and maintains period length of approximately 24 h in all cells in the organ more precisely than clocks in individual cells^12^.

Period circadian clock (Per) genes *Per1* and *Per2* are main components of the circadian clock feedback loop and have three important roles. First, they are essential for circadian oscillation, as demonstrated by the fact that *Per1*/*Per2* double-knockout mice become arrhythmic^13^. Second, *Per* genes are also important as a core component of the ruler of the circadian period^14^. Sequential phosphorylation, primarily by casein kinase 1 (CK1), at a large number of sites is important for this property and may constitute the actual posttranslational oscillator in mammalian circadian clocks^15^,^16^. Third, induction is a key factor of circadian entrainment. Light-induced expression of Per1 and Per2 proteins in the SCN appears to be important in entrainment^17^. However, the cooperative roles of Per1 and Per2, especially in determining circadian period length and under various light conditions, still remain to be elucidated. In this study, we found a new role, and its mechanism, of Per1-Per2 cooperation using new methods we established.

## Results

### *Per1* expression is associated with a short period and *Per2* expression is associated with a long period

**Figure 1:**
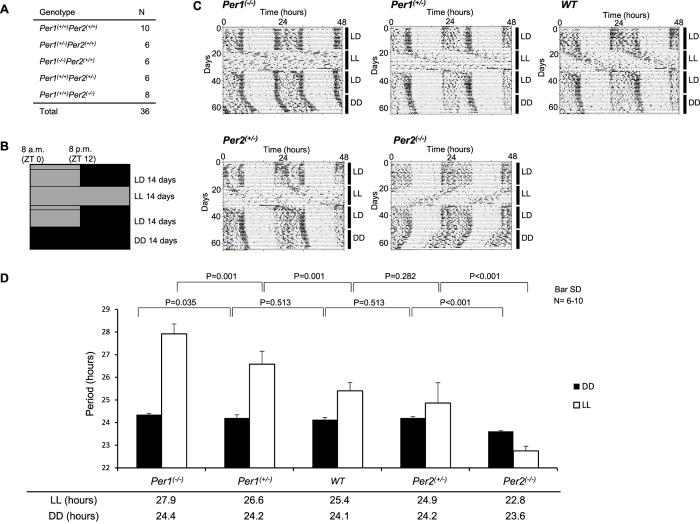
Period length under conditions of constant light depends on the ratio of *Per1* copy number to that of *Per2*. (**A**) Number and genotype of mice used in this experiment. (**B**) Schematic diagram of the experimental procedure. Animals were exposed to the indicated number of hours of light and/or dark for the indicated number of days. The light intensity was within the range of 100–300 Lux in the light phase at the centre bottom of the cage. (**C**) Representative ClockLab actogram for each genotype. (**D**) Period length determined by chi-square periodogram. Per1ldc and Per2ldc lines were used in this experiment. LD, light/dark; LL, light/light; DD, dark/dark; ZT, zeitgeber time. Values represent mean ± standard deviation (SD) of a single experiment. P-values were calculated using one-way ANOVA and Tukey’s post hoc tests. The F and P-values from the Tukey’s test are shown in Supplemental Table S2.

We first analysed the locomotor activity under conditions of constant light, which was reported to disrupt the coupling effects of the SCN^18^,^19^. We hypothesized that the free-running periods of *Per1*^(−/−)^ and *Per2*^(−/−)^ under conditions of constant dark are similar because SCN coupling maintains the circadian free-running period at 24 h in each genotype^20^,^21^. If constant light disrupts the SCN coupling mechanisms, we may be able to identify the period length from each cell and observe the cooperative roles of *Per1* and *Per2*. We analysed locomotor activity, based on infrared sensor counts of wild-type (WT), *Per1*^(+*/*−)^, *Per1*^(−/−)^, *Per2*^(+*/*−)^, and *Per2*^(−/−)^ mice under constant light conditions for 14 days (Fig. 1A,B). Mean period length in these animals is shown in Fig. 1C,D. We found that the period length of each genotype depended on the ratio of *Per1* copy number to that of *Per2*. That is, *Per1* expression is associated with a short period and *Per2* expression is associated with a long period.

We also performed experiments with Brd mice^22^, but no differences were noted between the heterozygous and homozygous *Per2* mutant mice (P = 0.93, Supplemental Table S1). Because we suspected that the dominant negative effects resulted from the deletion of the PAS domain^23^, we only utilized the ldc mouse line^13^ in subsequent studies.

### Arrhythmic changes are not likely to occur under conditions of constant light if either *Per1* or *Per2* are deficient

**Figure 2:**
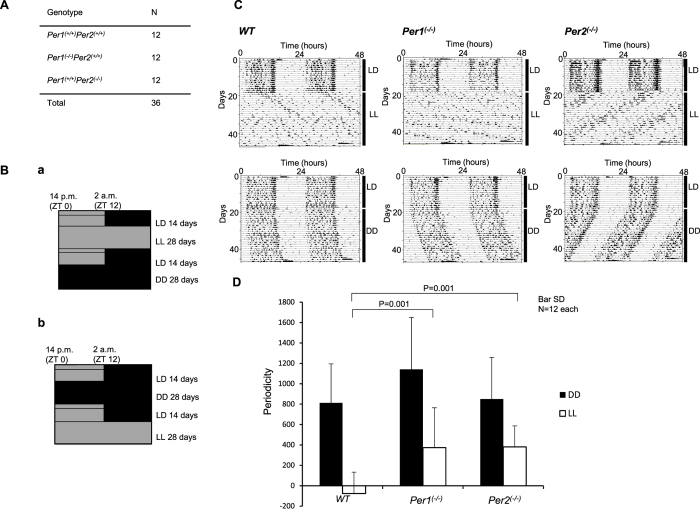
Arrhythmicity under conditions of constant light was not likely to occur when either *Per1* or *Per2* was deficient. (**A**) Number and genotype of mice used in this experiment. (**B**) Schematic diagram of the experimental procedure. Six mice of each genotype were used in each (a and b) crossover design experiment. Animals were exposed to the indicated number of hours of light and/or dark for the indicated number of days. The light intensity was within the range of 100–300 Lux in the light phase at the centre bottom of the cage. (**C**) Representative actogram for each genotype. (**D**) Periodicity is defined as the mean amplitude (Q[p]) minus the level of significance at the determined period length within ±1 h of the circadian period length, determined using the chi-square periodogram. Values represent mean ± standard deviation (SD) of a single experiment. P-values were calculated using one-way ANOVA and Tukey’s post hoc tests. The F and P-values from the Tukey’s test are shown in Supplemental Table S3.

Prolonged periods of constant light are known to cause arrhythmicity, which may mimic circadian disorders, including delirium. To observe the effect of constant light-induced arrhythmicity in *Per* knockout mice, we first synchronized the mice under conditions of a 12-h light/12-h dark cycle for 14 days. We then exposed WT *Per1*^(+*/*+)^*Per2*^(+*/*+)^, *Per1*^(−/−)^, and *Per2*^(−/−)^ mice (n = 12/group) to constant light for 28 days, analysed locomotor activities, and calculated the periodicity (Fig. 2A,B). Although WT mice gradually became arrhythmic with constant light for more than 14 days, *Per1*^(−/−)^ and *Per2*^(−/−)^ mice maintained rhythmicity (Fig. 2C,D). That is, arrhythmic change under conditions of constant light is not likely to occur if either *Per1* or *Per2* are deficient.

### *Per1* and *Per2* cooperatively confine the range of circadian period length to approximately 24-h

**Figure 3:**
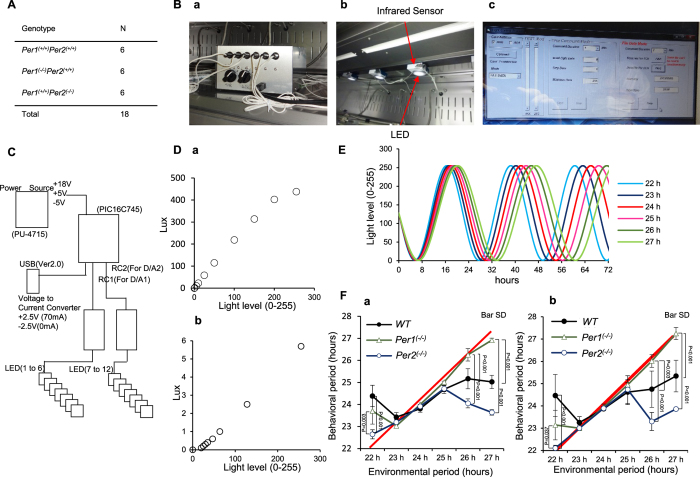
Establishment of the LED-based lighting system to generate any pattern of light intensity. Under conditions of gradually changing light, both *Per1*^(−/−)^ and *Per2*^(−/−)^ mice were entrained to a broader range of period length than WT mice. (**A**) Number and genotype of mice used in this experiment. (**B**) Components of 12-LED–based lighting system: (a) main unit, (b) infrared sensor and LED, (c) interface. (**C**) Block diagram of LED control device. (**D**) Light intensity (lux) measured by luminance meter on the floor of the rack just below the LED at each light level (0–255) with (a) normal and (b) 1/100 electric current. We used 1/100 current so that mice would not sense the light as constant. (**E**) Pattern of gradually changing light level programmed in this experiment. (**F**) Behavioural period (the circadian period of locomotor activity) determined by chi-square periodogram and environmental period (the period of the light cycle) under gradually changing light with period of 22 h to 27 h for (a) 2 weeks (b) final 1 week. We performed both chi-square periodograms for a duration of 2 weeks and one with a final week for each environmental period. The red line shows the concordance of environmental and behavioural periods. The light intensity was approximately 0.01 Lux at the trough and approximately 5.4 Lux at the peak at the centre bottom of the cage. P-values were calculated using one-way ANOVA and Tukey’s post hoc tests. The F and P-values from the Tukey’s test are shown in Supplemental Table S4.

We then performed experiments using T-cycles, patterns of regular alternation of light and dark with period different than 24 h, to mimic the experience of shift workers^24^,^25^. Although a previous study showed that WT, *Per1*^(−/−)^, and *Per2*^(−/−)^ genotypes have similar T-cycle properties^26^, we hypothesized that this is because of a rectangular-type light pattern, characterized by abrupt changes between light and dark, which induces strong and unnatural phase shifts. In fact, several studies showed that a gradually changing or natural pattern of light induces a different entrainment pattern than that induced by a rectangular-type light pattern^27^,^28^,^29^. We also hypothesized that we would be able to see endogenous circadian clock rigidity and flexibility in WT and *Per* knockout mice if we could create a system without strong phase shifts. To avoid initiating a phase shift by turning the lights on and off, we established a light-emitting diode (LED)-based lighting system that can generate any pattern of light intensity at 1-min resolution and produces 256 levels of light intensity (Fig. 3B–D; Supplemental Figure S1). We analysed locomotor activities of WT, *Per1*^(−/−)^, and *Per2*^(−/−)^ mice (Fig. 3A) under gradually changing light with periods of 22–27-h, in increments of 1 h (but not in that order), for 2 weeks each (Fig. 3E,F; Supplemental Figures S2 and S3). Behavioural period (the circadian period of locomotor activity) determined by chi-square periodogram and environmental period (the period of the light cycle) are shown in Fig. 3F. Under conditions of gradually changing light with period of 23, 24, or 25-h, the circadian rhythm of WT, *Per1*^(−/−)^, and *Per2*^(−/−)^ mice became entrained to the environmental light pattern. However, only that of *Per2*^(−/−)^ mice became entrained to the 22-h period, and only that of *Per1*^(−/−)^ mice became entrained to the 26- and 27-h periods. That is, both *Per1*^(−/−)^ and *Per2*^(−/−)^ mice were entrained to a broader range of period length than WT mice.

The results of these three sets of experiments suggest that *Per1* expression is associated with a shorter free-running period, *Per2* with a longer period, and that *Per1* and *Per2* cooperatively confine the range of circadian period length to approximately 24 h. Cooperation between *Per1* and *Per2* results in a rigid 24-h rhythm that is not perturbed by the environment. On the other hand, this system is relatively inflexible with regard to adapting to environmental change, which leads to morbid deterioration (i.e., arrhythmicity or lack of entrainment to new environmental conditions) under various light conditions.

### Establishment of *Per2* knockout-rescue system to detect period shortening in FASPS

Next, to analyse cooperative roles of *Per1* and *Per2* at the cell culture level, we established a *Per2* knockout-rescue system. Rescue of clock gene knockouts can be used to analyse many mutant phenotypes^30^, and several systems have been described for period genes, including one involving *Per2* knockout in mouse embryonic fibroblasts with adenoviral induction^31^, and another system employing stable transfection of NIH3T3 cells^32^. However, these systems failed to detect a change in period length, probably because period length is influenced by the amount of Per protein in each cell, and gene dosage is critical for *Per* genes^33^,^34^,^35^. The amount of Per protein in each cell should be tightly controlled at a low level (~1,000 molecules/cell)^36^,^37^. Therefore, we used a *Per2* knockout-rescue system with *Rosa26* targeting and embryonic stem (ES) cell differentiation because genes introduced into the *Rosa26* locus on murine chromosome 6 are expressed in every tissue in the body^38^,^39^. If we insert only one copy of the rescue gene cassette at *Rosa26*, we can compare the period length of each mutant precisely.

**Figure 4:**
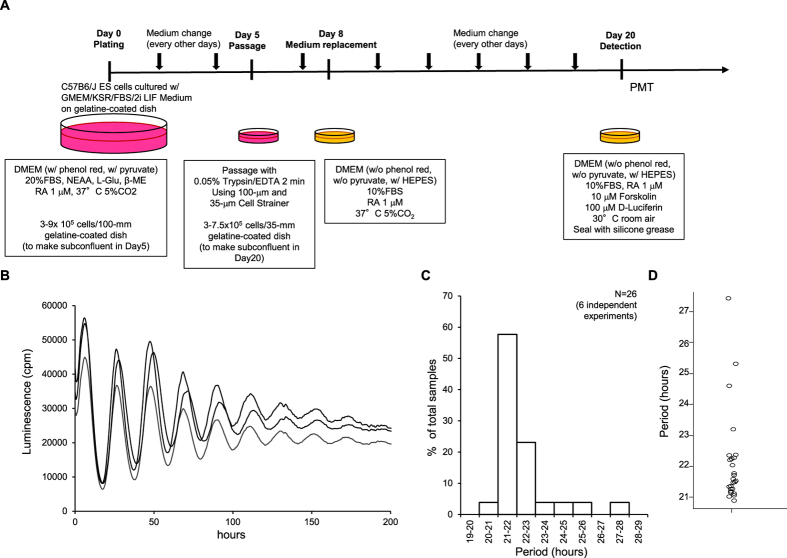
Establishment of the ES cell differentiation system to detect precise period length. (**A**) Schematic diagram of ES cell differentiation system. (**B**) Real-time monitoring of luminescence of *Per2::Luc KI/KI* ES cells on day 20 with photomultiplier tube; cpm, counts per min. (**C**) Histogram and (**D**) dot plot of results of autocorrelation analysis of period length using *Per2::Luc KI/KI* ES cells. Values in (**C**) are mean % of total samples of six independent experiments. Values in (**D**) represent individual samples in six independent experiments. GMEM, Glasgow Minimum Essential Medium; KSR, KnockOut Serum Replacement; FBS, foetal bovine serum; 2i, 2 inhibitors (CHIR99021 and PD0325901); LIF, leukaemia inhibitory factor; DMEM, Dulbecco’s Modified Eagle Medium; NEAA, Non-Essential Amino Acids; L-Glu: L-glutamine. β-ME: β-mercaptoethanol; RA, retinoic acid. Each line and dot indicates each 35-mm dish sample.

First, we focused on the fact that ES cells do not have a circadian rhythm, but circadian oscillation appears with retinoic acid (RA)-induced differentiation^40^, and we can see circadian oscillation in ES cells of any genotype without generating an organism. We performed a broad-parameter search to produce sustained oscillation with high amplitude to detect precise period length. Finally, we improved the ES differentiation system^40^ (Fig. 4A) and succeeded in detecting 10 peaks of oscillation in *Per2::luciferas*e *knockin (KI)/KI* ES cells (Fig. 4B)^41^. We also used this protocol in several independent experiments and observed reproducible, precise period length, that is, most of the cell culture dishes were included within approximately 1-h, although some outliers were apparent (Fig. 4C,D).

**Figure 5:**
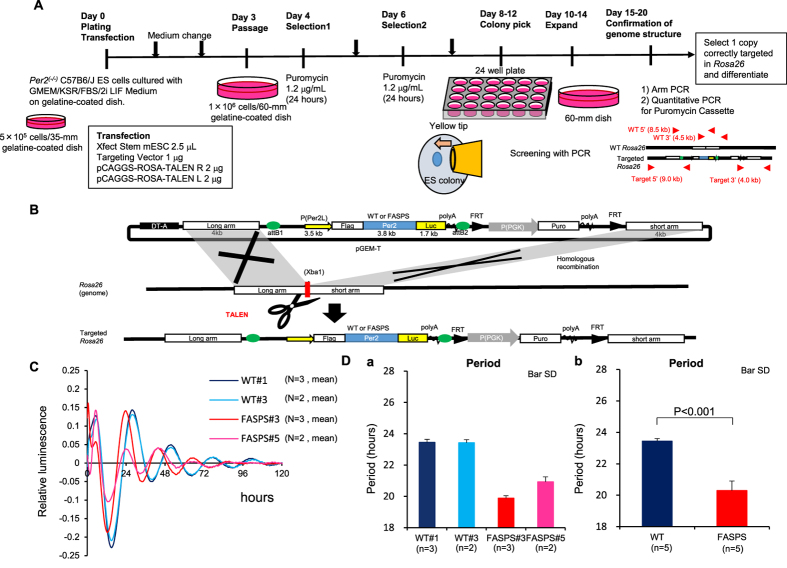
Establishment of *Per2* knockout-rescue system to detect period shortening in FASPS. (**A**) Schematic diagram of *Per2* knockout-rescue targeting. We introduced *Per2* into the *Rosa26* locus of *Per2*^(−/−)^ ES cells using TALEN, picked colonies, and checked genome structure with Arm PCR and quantitative PCR. ES clones containing only one copy of the rescue construct were differentiated. (**B**) Schematic diagram of homologous recombination between targeting vector and *Rosa26* locus. (**C**) Detrend/baseline oscillation of two independent lines each of WT and FASPS mutant ES cells. (**D**) Period length calculated by autocorrelation analysis in individual (a) lines and (b) genotypes. FASPS, familial advanced sleep phase syndrome; FRT, flippase recognition target; P(PGK), mouse phosphoglycerate kinase 1 promoter; DT-A, diphtheria toxin fragment A.

We then introduced *Per2* into the *Rosa26* locus of *Per2*^(−/−)^ ES cells using TAL effector nuclease (TALEN) (Fig. 5A). Specifically, we constructed a vector containing the *Per2* promoter (3.5 kb, P(Per2L)), *Per2* coding region, and luciferase gene between the *Rosa26* long arm (4 kb, modified from 8 kb) and short arm (4 kb) (Fig. 5B). We transiently cotransfected *Per2*^(−/−)^ ES cells with this vector and TALEN vectors^42^, which create a double-strand break in the *Rosa26* region, to improve recombination efficiency. Then we selected ES cells with puromycin and picked colonies under a phase contrast microscope. We checked the structure of the *Rosa26* locus by polymerase chain reaction (PCR) (targeted arm 5′, 3′, WT arm 5′, 3′) and quantitative PCR of the puromycin resistance gene (Supplemental Figure S4). We obtained several rescued *Per2* knockout ES cell lines that contained only one copy of the *Per2* gene cassette correctly introduced into *Rosa26*. By introducing the WT *Per2* gene, we were able to detect the 24-h oscillation from two independent ES clones (Fig. 5C,D, Supplemental Figure S5A).

Next, we used a familial advanced sleep phase syndrome (FASPS) mutant to verify this system. FASPS is an autosomal dominant disease characterized by a natural tendency to go to sleep and wake up at times considered earlier than normal. This phenotype is caused by period shortening of the circadian rhythm due to *Per2* point mutation^5^. This is the only known disorder caused by a *Per* gene mutation and can be modelled with *Per2* knockout mice rescued with a bacterial artificial chromosome (BAC) clone containing the *Per2* gene locus with the S659G mutation, which corresponds to the human S662G mutation^43^. We introduced the FASPS mutant into this system and detected period shorter than 24 h in two independent ES clones [WT (mean ± standard deviation [SD]): 23.4 ± 0.17 h, FASPS: 20.3 ± 0.6 h; P < 0.001] (Fig. 5C,D, Supplemental Figure S5A). To our knowledge, this is the first system to detect period shortening in a FASPS mutant at the cell culture level. Moreover, we also introduced two known mutants. The first, mut6, consists of six mutations of amino acids downstream of the FASPS mutation (S662A/S665A/S668A/S670A/S671A/T672A) that are said to be phosphorylated serially after phosphorylation of the FASPS site and thought to function in a manner similar to FASPS. In a previous study, mut7, which includes the FASPS site mutation, was used, but we omitted that mutation from this mutant^32^. The second known mutant introduced was S477A/G479A, a mutant of the β-transducin repeat-containing protein (β-TrCP) binding site^44^,^45^, which is the E3 ubiquitin ligase for Per proteins. Both mutants were used in a previous study, but period length was not determined. With introduction of the mut6 and β-TrCP mutants, period shortening similar to that observed with FASPS was observed for mut6 (20.3 ± 0.8 h) but period change was not observed in β-TrCP mutants (24.1 ± 0.8 h) (Supplemental Figure S6). These data demonstrate that our *Per2* knockout-rescue system can produce the phenotypes of other *Per2* mutations.

### The coding regions appear to be important in the difference between *Per1* and *Per2*

**Figure 6:**
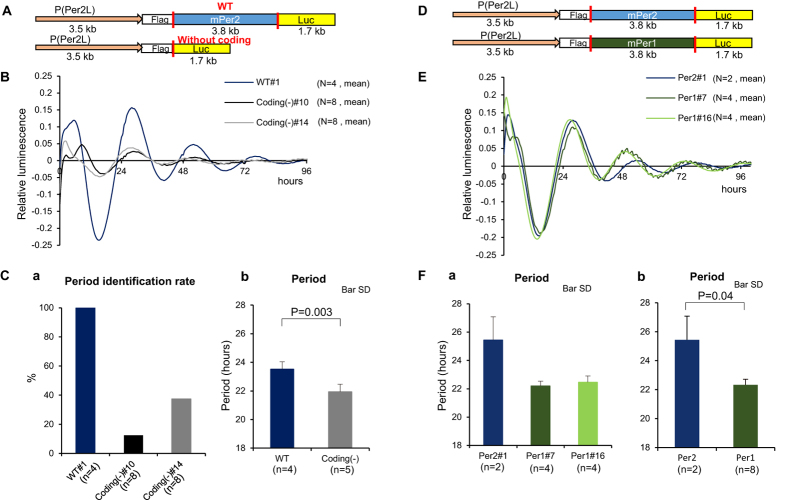
Demonstration of *Per1*-*Per2* cooperativity at the cell culture level. The presence of the coding regions, not the promoters, seems to be important in the difference between *Per1* and *Per2*. (**A–C**) Comparison of *Per2* knockout cells rescued with and without *Per2* coding region. (**A**) Rescue constructs with or without *Per2* coding region. (**B**) Detrend/baseline oscillation of ES cells rescued with or without *Per2* coding region. Results of analysis of two independent experiments are shown. Data with *Per2* coding region were generated using one cell line (n = 2, twice). Data without coding region were generated using two lines (n = 2, 2 lines * 2 conditions, twice). (**C**) Rate of oscillation period identification (a) and comparison of period length (b). (**D–F**) Comparison of rescue with *Per2* and *Per1* coding regions. (**D**) Rescue constructs containing *Per2* or *Per1* coding region. (**E**) Detrend/baseline oscillation of ES cells rescued with *Per2* or *Per1* coding region. (**F**) Comparison of the period length calculated by autocorrelation analysis in individual (a) lines and (b) genotypes. The P-value was calculated using the Mann Whitney U test.

**Figure 7:**
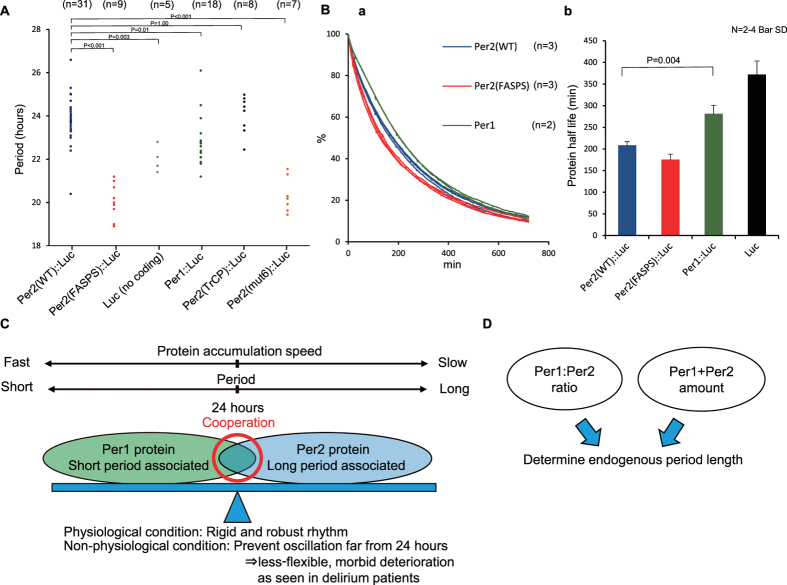
Models and hypotheses of period length and protein half-life. (**A**) Data from all rescued *Per2* knockout ES cells are shown. Each point represents one 35-mm dish. These data include outliers, but not dishes for which oscillation was not determined. P-values were calculated using one-way ANOVA and Tukey’s post hoc tests. The F and P-values from the Tukey’s test are shown in Supplemental Table S5. (**B**) Degradation curve (a) and half-lives (b) of Per2(WT), Per2(FASPS), and Per1 are plotted against time. (**C**) Model of short period-associated Per1 and long period-associated Per2 proteins. (**D**) Our hypothesis on the factors that determine endogenous period length. Period length at a greater concentration of Per2 (e.g. *Per1* knockout) is longer than the period length observed at a greater concentration of Per1 (e.g. *Per2* knockout) because the degradation rate of Per2 is higher and its rate of accumulation is slower than Per1. The study by Gerard *et al*.^82^ that modelled the negative autoregulation of gene expression showed that the period increases with an increased degradation rate when degradation is described by a Michaelis-Menten function. This was in accord with the results of the current study that indicated a difference in the period length and half-lives of Per1 and Per2.

Because our system proved to be versatile, we also analysed cells transfected with the construct without the *Per2* coding region (Fig. 6A). Oscillation was weaker and period was shorter than in cells expressing WT *Per2* (Fig. 6B,C, Supplemental Figure S5B). This suggests that cooperative roles of *Per1* and *Per2* are also important at the cell culture level. Next, we replaced the *Per2* coding region in the construct with that of *Per1* (Fig. 6D). Likewise, we utilized western blotting to examine Per1 and Per2 expression in Per1- and Per2-rescued ES cells. Both ES cell lines lacked native Per2 but expressed similar levels of native Per1. As expected, Per1::Luc was only expressed in the Per1-rescued ES cells, and Per2::Luc was only expressed in the Per2-rescued ES cells (Supplemental Fig. 7). *Per1* and *Per2* have different promoter structures, mRNA expression patterns, and mRNA stability controlled by the 3′ UTR^17^,^46^,^47^. Moreover, in the *Per2* knockout-rescue system with adenoviral induction described in a previous report, *Per1* and *Per2* coding regions showed a similar effect on oscillation, although period was not determined^31^. Therefore, we expected to see similar periods with this experiment. Contrary to our expectations, we observed period shortening with Per1 expression (22.3 ± 0.4 h) compared to Per2 (25.4 ± 1.6 h) in the two independent ES clones (Fig. 6E,F, Supplemental Figure S5C). That is, the association of *Per1* expression with a shorter period and that of *Per2* with a longer period is at least partly explained by differences in the *Per* coding regions, and not the *Per* promoters as previously reported^31^. We also analysed data from all ES cell experiments (Fig. 7A). The period length could be divided into three groups. WT and β-TrCP mutant lines had periods of approximately 24 h. Cells expressing constructs in which the *Per2* coding region was lacking or replaced by that of *Per1* had periods of approximately 22–23 h, and the FASPS and mut6 mutants had periods of approximately 20 h.

### Per1 has a paradoxically longer half-life than Per2

Until now, the effects of Per1 and Per2 on circadian oscillation were thought to be similar, and specific properties of the two proteins were not a focus. To analyse the mechanism underlying differences in period length associated with Per1 and Per2 expression, we calculated protein half-life. Several groups reported that FASPS mutants have shorter half-lives than WT Per2, and Per protein half-life and period length are believed to be correlated^32^,^48^,^49^. To analyse the half-lives, we transfected NIH3T3 cells with luciferase fusion proteins, treated the cells with cycloheximide, and conducted real-time monitoring of bioluminescence^14^. We chose this approach for two reasons. First, the bioluminescence system has a broad dynamic range, and it is possible to quantitate small differences in protein level with sensitivity. Second, with real-time monitoring, we can track the same cell population and compare luminescence in the same cells at different time points to calculate the half-life. Using this approach, we observed a shorter half-life of the FASPS mutant, but Per1 had a paradoxically longer half-life than Per2 (Fig. 7B, Supplemental Figure S8A). Because we wanted to show that the observed difference in half-life was not just the consequence of phase difference, we also performed the experiments under dispersed circadian phase conditions^14^ and obtained clearer results (Supplemental Figure S8B). We also compared the half-lives of Per1 and Per2 by western blotting without the luciferase fusion (Supplemental Figure S8C,D) and observed that Per1 appeared to be more stable (P = 0.01). Likewise, we demonstrated that period length and protein half-life are not always correlated (Supplemental Figure S9A). These findings suggest that the mechanisms underlying period shortening in FASPS and underlying the association of Per1 expression with a short period are different.

## Discussion

In this study, we have shown that *Per1* expression is associated with a short period and *Per2* expression with a long period, and this is explained, at least in part, by differences in the properties of the two proteins, and not the *Per* promoters as previously reported. Per1 and Per2 cooperatively confine the circadian period to 24 h, and prevent the period length from oscillating far from 24 h. However, this system is relatively inflexible and deteriorates readily, i.e., does not become entrained to the environmental cycle or becomes arrhythmic under various light conditions (Fig. 7C). With regard to methodology, we established new experimental methods to analyse the cooperativity of Per1 and Per2. First, we established an LED-based lighting system that can generate any pattern of light intensity to measure oscillation period at the organismal level. Next, we established an ES cell differentiation system to precisely measure period length at the cell culture level. Finally, we established a Per2 knockout-rescue system that is the first to recapitulate the period shortening seen in FASPS at the cell culture level. Using these new methods, we have clearly shown the importance of Per1-Per2 cooperativity at the organismal and cell culture levels.

We have shown that cooperation between Per1 and Per2 results in rigid circadian oscillation and confines the circadian period to approximately 24 h. Until relatively recently in evolutionary history, most mammals lived in the same general area on the earth; there was little artificial light, and they had to adjust their circadian clocks only to the 24-h environmental cycles. Thus, a rigid circadian rhythm maintained through Per1-Per2 cooperativity would be beneficial for survival, to predict the day-night transition unperturbed by environmental noise^50^. However, in modern society, artificial lighting is nearly ubiquitous, producing conditions of constant light similar to those used in the experiments described in Figs 1 and 2. Long airline flights and systems employing shift workers are also features of modern life, and generate conditions such as cycles of different period length observed with gradually changing light in the experiments described in Fig. 3.

Recent reports have suggested that delirium is one of the most common^51^,^52^ circadian disorders of the hypothalamus^53^,^54^,^55^. Delirium is characterized by the onset of sudden and severe confusion and occurs in more than 30% of aged or dementia inpatients. Although delirium is defined as a transient and reversible disturbance of attention and cognition, one of the most common symptoms of delirium is sleep-wake cycle disorder, or day-night reversal, or arrhythmicity^6^,^53^,^54^,^55^. One study^6^ showed that 97% of delirium patients suffered from sleep-awake cycle disturbance and attention disturbance. Further, delirium is a serious concern in the geriatric field because it causes many in-hospital accidents, including falls and fractures^56^. However, the results of a randomized controlled trial have shown that delirium is treatable with chronobiological therapies^57^,^58^, including ramelteon, a melatonin receptor agonist^57^. Hence, circadian disorders are a core symptom and perhaps one of the primary causes of delirium.

We have attempted to mimic the conditions of circadian disorders, including delirium, using constant and gradually changing light in our experiments. One of the major causes of delirium in patients in intensive care units is the constant light, as in the experiments described in Figs 1 and 2. WT mice in these experiments may have experienced a condition similar to delirium. Under these conditions, a rigid circadian rhythm maintained through Per1-Per2 cooperation would negatively affect humans and result in morbid deterioration, similar to that observed in mice in the experiments described in Figs 2 and 3. As shown in Fig. 3, *Per* knockout mice were not likely to develop day-night reversal; thus, we think that the independent regulation of Per1 and Per2 expression is a potential drug target for the treatment of circadian disorders. Nonetheless, we cannot mimic the symptoms of delirium, because there are no experimental systems using mice to model the other characteristic symptoms of delirium, including disorientation or illusion and delusion, which are related to consciousness.

The question of whether *Per* knockout mice are resistant to “day-night reversal” in “non-gradually changing light” or a “rectangular-type light pattern” has been addressed in three previous studies. Although one prior study showed that WT, *Per1*^(−/−)^, and *Per2*^(−/−)^ mice have similar T-cycle entrainment patterns^26^, a smaller proportion of WT mice were entrained at the T21 T-cycle compared to *Per2*^(−/−)^ mice, which was similar to our observations (Fig. 3). Next, there is the extreme case of *Per1*^(−/−)^*Per2*^(−/−)^ double-knockout mice that never progress to the state of day-night reversal, not even when exposed to the 12 h LD cycle or any period cycle, which is called masking^59^. Third, our findings that *Per1*^(−/−)^ and *Per2*^(−/−)^ mice could be adapted to a broader range of periods under various T-cycles was in accord with previous findings in which *Per2/Cry2* and *Per1/Cry1* double knockout mice entrained to the 16–32 h LD cycle^60^. However, our findings varied slightly because the T-cycle entrainment patterns of *Per1* and *Per2* knockout mice were different under gradually changing light.

Although jet-lag experiments are often used to mimic patterns experienced by shift workers^61^, we used T-cycles^24^,^25^, which can also reproduce some features of the shift worker environment. The two types of shift worker rotations are forward and backward^24^. The forward shift is associated with a regularly delayed phase of the sleep-wake cycle (e.g. the morning shift, then the afternoon shift, then the night shift) that resembles a greater than 24-h entrainment cycle, whereas the backward shift is the reverse. Forward shifts are often used because a shift will generate rhythms that coincide with most rhythms of the human free-run cycles^25^,^62^.

With regard to the intrinsic period of *Per1*^(−/−)^ and *Per2*^(−/−)^ cells, a detailed predictive modelling study^63^ showed that *Per1*^(−/−)^ knockout cells have a longer period whereas *Per2*^(−/−)^ knockout cells have a shortened period, which was compatible with the majority of our findings, including our protein stability results. However, a number of studies have reported diverse results^12^,^64^,^65^,^66^, which could be due to the fact that bioluminescence experiment results are difficult to interpret, and are substantially dependent on long culture conditions, reporter differences, and period calculation methods. The results from organ culture might also reflect the complicated aftereffects^67^,^68^ and the period modification apparent following intercellular signal transduction in heterogeneous cells^69^, which could be greater in 3D organ culture than in 2D cultured cells. Complicated aftereffects and the interaction of heterogeneous cells might also cause tissue-specific differences^64^,^66^. Thus, organ culture might not accurately reflect the period length of the intracellular molecular machinery.

Although a former study demonstrated that *Per2*^(−/−)^ cultured cells are arrhythmic^12^, we found that *Per2*^(−/−)^ cells had a shorter period similar to *Per1::Luc*-rescued ES cells [Figs 6Cb and 7A Luc (no coding)]. However, the oscillation of the *Per2*^(−/−)^ cells was weak and unstable, and we could only detect the oscillation in cell culture under optimal conditions, which was consistent with the prior results. The former study^12^ also showed that *Per1*^(−/−)^*Per2::Luc* cultured cells are arrhythmic, which could indicate that the period length of *Per1*^(−/−)^ cells was too long to detect periodicity, although we have to be cautious considering that the luciferase fusion appears to diminish the function of Per2 to some extent (Fig. 4). Nonetheless, our data and the results of former studies indicate that *Per1*^(−/−)^ cells have a longer intrinsic period and *Per2*^(−/−)^ cells have a shorter period, although both are unstable in the absence of intercellular synchronization.

Under constant dark conditions, *Per1* or *Per2* knockout mice had a free-running period of approximately 24 h, but the circadian period was dramatically different under the LL condition, in that *Per1*^(−/−)^ mice displayed a long period, whereas *Per2*^(−/−)^ mice exhibited a short period^22^ (Fig. 1). Hence, we confirmed the prior results, although the results were slightly different, most likely due to the construct difference between the Brd and the ldc mice.

Previous studies have likewise shown that the ratio of the phase advance portion to the phase delay portion of the phase response curve (PRC) accurately predicts the lengthening of the activity rhythm period in LL^26^ in *Per1*^(−/−)^ and *Per2*^(−/−)^ mice. However, the molecular mechanisms involved have not yet been elucidated. In our studies, we examined *Per1*^(+*/*−)^ mice and *Per2*^(+*/*−)^ mice and found that the ratio of the *Per1*:*Per2* copy number was important to the period length in the LL condition. A possible explanation is that the light-dependent induction of Per proteins enlarged the difference in the accumulation rate between Per1 and Per2 proteins^17^,^70^.

Another possible hypothesis is the occurrence of intercellular uncoupling in LL. Many studies involving clock gene mutant mice have shown that although there are some exceptions, the period length in animals is closer to 24 hours than the period length observed at the cell culture or organ level^12^,^30^,^49^,^71^,^72^, most likely due to the synchronization mechanism of coupling. As previously discussed, we believe that *Per1*^(−/−)^ cells have a longer intrinsic period and *Per2*^(−/−)^ cells have a shorter period; however, the period length in animals could be close to 24 hours due to SCN coupling in the DD condition. Further, conditions of constant light might induce the uncoupling of SCN neurons, allowing the endogenous cellular period length to surface^18^,^19^. Finally, this uncoupling hypothesis does not necessarily exclude the phase response curve hypothesis, but these might be two aspects of the same phenomenon.

In this study, we have shown that the *Per1/Per2* ratio is important for period length, but we have not assessed the effect of total Per protein level. Previous studies have suggested that both total Per protein level and *Per1/Per2* ratio are important in endogenous period determination^33^,^35^,^71^. Our hypothesis regarding the determination of endogenous period length is shown in Fig. 7D. A previous immunohistochemical analysis revealed that *Per1*^(−/−)^ mice have lower Per2 protein levels than WT mice, and *Per2*^(−/−)^ mice have lower Per1 protein levels in the SCN than WT mice, which might be the result of a mechanism that balances the ratio of Per1:Per2^13^. Future studies involving western blotting of the SCN could lead to a more refined conclusion; however, at least 10 animals of each genotype^73^ would be necessary for each SCN western blotting assay, and additional animals would be required for the clock proteins.

Utilizing western blotting to examine Per1 and Per2 expression in Per1- and Per2-rescued ES cells, we have shown that both ES cell lines lacked native Per2 but expressed similar levels of native Per1, that Per1::Luc was only expressed in the Per1-rescued ES cells, and that Per2::Luc was only expressed in the Per2-rescued ES cells. However, we could not determine the ratio of Per1 and Per2 because the Per1 and Per2 antibodies are different, and the transfer efficiency of the native protein and the luciferase-fusion protein from the acrylamide gel to the membrane varied due to a 60 kD difference in the molecular weights of the proteins. One might assume that measuring the total amount of the native (e.g. Per1) and the luciferase-fusion protein (e.g. Per1::Luc) is possible using the standard purified form of each protein. However, in the current study, we had to determine the absolute concentration of each standard protein using Coomassie Brilliant Blue (CBB) stain. Because each protein stain has a preference for specific amino acids (e.g. basic amino acids in the case of CBB), the native and luciferase-fusion proteins were not comparable^74^. Thus, it appears impossible to compare the ratio of Per1 and Per2 by western blotting using a mixture of the native protein (e.g. Per1) and the luciferase-fusion protein (e.g. Per1::Luc). Future studies involving mass spectrometry and the use of common peptides from the native and luciferase-fusion proteins as standards could enable the measurement of the Per1:Per2 ratio in these ES cells^75^.

Half-life analysis showed that Per1 has a paradoxically longer half-life than Per2. This difference in half-life may have important consequences for the mechanism(s) underlying the association of Per1 expression with a short period and of Per2 with a long period, which is independent of FASPS period shortening. Our hypothesis is shown in Supplemental Figure S9B. Degradation of Period proteins is thought to occur at the fully phosphorylated state. In the case of the FASPS mutant, these proteins are thought to quickly become fully phosphorylated^76^,^77^ and overall half-lives would become shorter^77^,^78^,^79^. Because Per1 and Per2 have similar phosphorylation sites, including the FASPS site, we think the difference would come, not from the rate of serial phosphorylation, but from the rate of protein degradation itself. In the case of Per1, degradation is slower and accumulation is quicker; then, early negative feedback occurs, leading to shorter circadian period length. In the case of Per2, degradation is faster and it accumulates for a longer time. This hypothesis is consistent with previous simulation analyses^80^,^81^. Another theoretical study of the negative autoregulation of circadian gene expression also showed that the period increases with an increased degradation rate when degradation is described by the Michaelis-Menten function^82^. Thus, an enzyme sequestration mechanism might be important for aligning the Per1 and Per2 cycling rates^16^. The results of a detailed predictive modelling study of the Per1 and Per2 proteins were likewise consistent with our findings and our model (Fig. 7C and Supplemental Figure S9B)^63^. This detailed predictive modelling study showed that *Per1*^(−/−)^ knockout cells have a longer period and *Per2*^(−/−)^ knockout cells have a shortened period. In addition, this model indicated that Per2 is degraded more quickly than Per1. Moreover, the peak phase of the Per1 protein was earlier than the Per2 peak phase, which is consistent with the notion that Per1 accumulates more rapidly, as indicated by our results. However, these models represent only one possible explanation of our results; thus, future studies that focus on the mechanisms underlying the differences between Per1 and Per2 are required.

In this study, we used clock protein-luciferase fusion proteins, which are widely used and reflect endogenous degradation^14^,^83^. Moreover, the behaviour and function of these fusion proteins are generally consistent with endogenous proteins^84^. Although we assumed that Per2 and Per2::Luc would have slightly different functions, we believed the comparison between Per1::Luc and Per2::Luc would be valid. Because the C-terminal end is longer in Per1 than Per2^17^, the possibility exists that the differences in the half-lives of Per1 and Per2 could simply be due to different effects of the luciferase on the stability of the Per1 and Per2 proteins. However, this possibility is not likely because a prior study (Fig. S5B; Isojima *et al*.^14^) showed that Luc::Per1 is more stable than Luc::Per2 in HEK 293 cells. Interestingly, the differences in the half-lives were apparent when the CK1ε vector was cotransfected. Therefore, CK1ε might play a role in the difference in the half-lives of Per1 and Per2.

Two non-covalent (weak) bonds existed between Per proteins and the peroxidase used for western blotting due to the use of primary and secondary antibodies. In contrast, only covalent (strong) bonds existed between the Per proteins and luciferase in the fusion protein assay. In addition, western blots involve lysed and dead cells, whereas living cells are continuously used in luminescence assays. Thus, we felt that the fusion protein assay was the more direct and powerful approach for assessing protein concentration.

In conclusion, we have shown that short period-associated Per1 and long period-associated Per2 proteins cooperatively confine circadian period to “*circa*” 24 h, which is rigid and relatively inflexible.

## Materials and Methods

Extended details of the materials and methods are described in the Supplemental Methods, including the recombinant plasmid production, the animals and the behavioural analysis, the LED-based lighting system, genotyping, the ES cells and cell culture conditions, methods for gene targeting and colony picking, the PCR screening, the Arm PCR, the quantitative PCR for confirmation of copy number, the ES cell differentiation system used to detect the precise circadian period length, the measurement of Per1 and Per2 half-life, western blotting, and other statistical analyses. Below is an abbreviated description of the major experimental procedures.

### Recombinant Plasmid Production

With the *Per2* promoter, we refer to the distal (3518 bps) promoter as the *Per2* long promoter (P(Per2L)). Luc(c) refers to the luciferase gene inserted into the PI-Psp1 site and positioned to fuse with the coding region for the C-terminal end of the gene of interest.

The pCMV-Sport2 mPer1 plasmid was kindly provided by Cheng Lee (Baylor College of Medicine) through AddGene^85^. The pTVCI2-DT-RareCutSite-ROSA 4 kb arm-Gateway <attR1/2> -FRT<P(PGK)–puro–polyA >–ROSA short arm 4 kb plasmid and the pCR8-P(Per2L)-Luc(c) plasmid were kindly provided by Maki Ukai-Tadenuma (RIKEN). The pMU2-Per2, pMU2^86^, pCAGGS-ROSA-TALEN-N153C63-R, and pCAGGS-ROSA-TALEN-N153C63-L plasmids were kindly provided by Hideki Ukai (RIKEN).

The experimental procedures used to construct the other plasmids are described in the Supplemental Methods. The coding regions of all of the plasmids used in this study were validated by full sequencing.

### Animals and Behavioural Analysis

All animal experiments were performed at the Center for Developmental Biology and were approved by the Animal Care Committee of the Center for Developmental Biology (approval ID: AH15-10-22) and all experimental procedures were performed according to the guidelines of the Animal Care Committee of the Center for Developmental Biology. We used *Per1* and *Per2* knockout mice from the ldc C57B6/J line^13^. *Per1*^(−/−)^*Per2*^(−/−)^ males (kindly provided by Genshiro A. Sunagawa of RIKEN) were mated with WT *Per1*^(+*/*+)^*Per2*^(+*/*+)^ C57B6/J females (Japan SLC) to generate F1 *Per1*^(+*/*−)^*Per2*^(+*/*−)^ mice for balancing genetic backgrounds. Next, we mated the F1 mice, obtained F2 mice, and performed genotyping. We used mice from each of the following genotypes: *Per1*^(+*/*+)^*Per2*^(+*/*+)^, *Per1*^(+*/*−)^*Per2*^(+*/*+)^, *Per1*^(−/−)^*Per2*^(+*/*+)^, *Per1*^(+*/*+)^*Per2*^(+*/*−)^, and *Per1*^(+*/*+)^*Per2*^(−/−)^. All mice used in the behavioural analysis were male and were greater than 8 weeks of age.

The animal behaviour analyses were performed using a behavioural analysis rack (Nihon-Ika) as previously described^87^. These racks are a special order product based on Clean Rack (Nihon-Ika, CR-1600S) and are made specifically for exposing mice to light-dark conditions.

The periodicity shown in Fig. 2 is defined as the mean amplitude (Q[p]) minus the level of significance at the determined period length within ±1 h of the circadian period length, determined using the chi-square periodogram. We calculated the periodicity for weeks 3 and 4 after initiation of constant light conditions.

### LED-based lighting system

An LED-based lighting system containing 12 lights was developed. This system uses the proportionate relationship between light strength and electric current. Linear lighting power (256 levels) was produced using a combination of eight resistance units. In this system, 12 mice can be exposed to any pattern of light intensity, with 256 levels at 1-min resolution to 12 mice, e.g., 6 mice exposed to two patterns each. The lighting program was used with the following formula: *I* = 128 + [0.5 + *A sin*2*π*(*x*_*min*_/(*T*_h_*60))], where *I* represents light intensity level (integer from 0–255); *A* represents amplitude of light intensity level (we used 127); *T*_*h*_ represents period of environmental light cycles (we performed the experiment using periods of 22–27 h, in increments of 1 h, (but not in that order) for 2 weeks each); and *x*_*min*_ represents the number of minutes after the start of the experiment. For the chi-square periodogram, more than 10 days are desired to avoid short-term random variations^88^. However, the transient or aftereffects were quite large in the first week because we could not connect the phase of each environmental cycle in these experiments. Hence, we performed both chi-square periodograms for a duration of 2 weeks and one with a final week for each environmental period.

Light intensity levels of 0–255 on the floor of the rack just below the LED were monitored using a CL-200A Chroma Meter (Konica Minolta) and changed almost linearly. Resistance of the main unit can be increased to 10× and 100×, i.e., 1/10 and 1/100 of electric current and lighting strength, respectively. So as not to make mice sense conditions of constant light^27^, we chose 256 levels from approximately 0.01 to 5.4 lux.

### Genotyping

Genotyping of *Per1* and *Per2* knockout mice was performed as previously described^13^ with the modification in the Supplemental Methods.

### ES cells and culture

*Per2::Luciferase KI/KI (Per2::Luc KI/KI*) ES cells^41^ were kindly provided by Etsuo A. Susaki (University of Tokyo). *Per2*^(−/−)^ ES cells were established by Naoshi Koide (RIKEN) from mice generated in this study.

ES cells were cultured in the absence of feeder cells in 60-mm dishes (Falcon) that had been coated with 0.1% autoclaved porcine skin gelatine (Sigma). Cells were cultured in ES medium consisting of Glasgow Minimum Essential Medium (GMEM, Gibco), 10% KnockOut™ Serum Replacement (KSR; Gibco), 1% foetal bovine serum (FBS), 1 mM sodium pyruvate (Gibco), 1 × MEM Non-Essential Amino Acids (Gibco), 100 μM β-mercaptoethanol (Wako, β-ME). Then, two inhibitors (2i), 3 μM CHIR99021 (Axon) and 1 μM PD0325901 (Wako) were added. ESGRO mouse leukaemia inhibitory factor (mLIF, Millipore) was added to a final concentration of 2 × 10^3^ U/mL. Cells were passed every other day onto 60-mm culture dishes.

### Gene targeting and colony picking

On day 0, *Per2*^(−/−)^ ES cells (5 × 10^5^) were plated in 2.0 mL ES medium 2i(+)LIF(+) on 35-mm culture dishes (Falcon) that had been coated with 0.2% gelatine. Five hours after plating, cells were transfected with 1 μg targeting vector, 2 μg pCAGGS-ROSA-TALEN-N153C63-R, and 2 μg pCAGGS-ROSA-TALEN-N153C63-L using Xfect Stem mESC reagent (Clontech) according to the manufacturer’s protocol with the modifications described in the Supplemental Methods. On day 3, cells were harvested and seeded at 1 × 10^6^ on 0.2% gelatine-coated 60-mm dishes. Selection with 1.2 μg/mL puromycin (Sigma) was carried out for 24 h on days 4 and 6. ES clones were picked on or after day 8 as described in the Supplemental Methods. Cells were plated in ES medium 2i(+)LIF(+) on 0.2% gelatine-coated 24-well plates (Techno Plastic Products). Approximately one third of the cells were lysed and used in PCR screening.

### PCR Screening

Pellets of picked ES cell colonies were treated with proteinase K (Roche). After centrifugation, supernatant was used in PCR to amplify the 3′ arm of the targeted allele (4.0 kb). Primer sequences and PCR conditions are described in the Supplemental Methods.

### Arm PCR

The integrity of the genome of targeted ES cells was confirmed by Arm PCR instead of southern blotting. To confirm homologous recombination of the targeting vector to the correct region of the genome, three PCRs were carried out: target 5′ (9.0 kb), WT 5′ (8.5 kb), and WT 3′ (4.5 kb). Primer sequences and PCR conditions are described in the Supplemental Methods.

### Quantitative PCR for copy number confirmation

Copy number of the inserted cassette and random integration were confirmed by quantitative PCR. The puromycin resistance gene was quantified and normalized to the level of TATA box-binding protein (TBP). The primer sequences and PCR conditions are described in the Supplemental Methods. The absolute levels of the PCR products were quantified using a standard curve. We analysed at least two lines of targeted clones that had only a single cassette inserted in the *Rosa26* locus.

### ES cell differentiation system to detect precise circadian period length

Cells were cultured from day 0 to day 8 in differentiation medium: Dulbecco’s Modified Eagle Medium (DMEM, high glucose, pyruvate, Gibco) containing 20% FBS, 0.1 mM MEM Non-Essential Amino Acids (Gibco), 100 U/mL penicillin and 100 μg/mL streptomycin (Gibco), 2 mM L-glutamine (Gibco), 100 μM β-mercaptoethanol (β-ME), and 1 μM RA (Sigma-Aldrich). On day 0, 3–9 × 10^5^ ES cells were plated in 10 mL differentiation medium on 0.2% gelatine-coated 100-mm dishes and cultured at 37 °C in a humidified atmosphere of 5% CO_2_. On day 5, cells were washed with DPBS without calcium and magnesium, incubated with 0.05% trypsin/0.48 mM EDTA (Gibco), and pipetted up and down in FBS. Cells were then sequentially applied to a 100-μm Cell Strainer (BD Falcon) and 35-μm Cell Strainer (BD Falcon). DMEM containing 10% FBS and 1× penicillin/streptomycin (Gibco) was added, followed by centrifugation. Supernatant was removed and cells were resuspended. 3–10 × 10^5^ cells were plated in 2 mL differentiation medium on 0.2% gelatine-coated 35-mm dishes (Falcon). On day 8, medium was changed to detection medium (DMEM without phenol red, without pyruvate, with 25 mM HEPES; Gibco) supplemented with 10% FBS, 1× penicillin/streptomycin (Gibco), and 1 μM RA. The medium was changed every other day. On day 20, the medium was changed to detection medium containing 1 μM RA, 10 μM forskolin, and 100 μM luciferin. Plates were sealed with silicone grease (Toray), and circadian oscillation was measured in a low-level light-detection unit (Hamamatsu photonics) at 30 °C in air.

The autocorrelation analysis of the circadian period was carried out using Mathematica 9.0 (Wolfram) as previously described^89^, with slight modifications as described in the Supplemental Methods.

### Measurement of Per1 and Per2 half-life

NIH3T3 cells (ATCC) were maintained in DMEM, high glucose supplemented with 10% FBS, 100 U/mL penicillin, and 100 μg/mL streptomycin (Gibco). For half-life measurement, cells were plated at 2 × 10^5^ cells/35-mm dish on day 0. On day 1, cells were transfected with 1 μg pMU2- Per1::Luc or pMU2-Per2::Luc using FuGene6 (Promega) according to the manufacturer’s protocol. On day 2, DMEM supplemented with 10% FBS, 100 U/mL penicillin, and 100 μg/mL streptomycin was added to each dish. On day 4, medium was changed to DMEM, high glucose (HEPES, no Phenol Red, Gibco) supplemented with 10% FBS, 100 U/mL penicillin, 100 μg/mL streptomycin, 100 μM luciferin (Promega), and 400 μg/mL cycloheximide (Wako). Plates were sealed with silicone grease (Toray) and luminescence was measured with a low-level light-detection unit (Hamamatsu photonics). Exponential fitting to calculate half-life was performed with R (R Development Core Team) and the code is detailed in the Supplemental Methods.

### Western Blotting

Western blotting was performed as previously described^90^,^91^,^92^ with slight modifications. The primary antibodies used in this study included anti-Mouse PER2 (Rabbit; PER21-A, Alpha Diagnostic International), anti-Per1 (Mouse), pAb (Guinea pig, PM091, MBL), monoclonal ANTI-FLAG M2 antibody produced in mice (Sigma F1804-50UG)), monoclonal anti-β-actin antibody produced in the mouse clone AC-15, ascites fluid (Sigma A5441). All of the antibodies were diluted in Tris-buffered saline solution/0.1%Tween (TBST) containing 5% skim milk at 4 °C. Each primary antibody was used at a 1000× dilution except for β-actin, which was used at a 5000× dilution. The secondary antibodies used in this study included rabbit anti-guinea pig IgG (H+L) secondary antibody, HRP-conjugate (Invitrogen 61-4620), ECL™ anti-mouse IgG, HRP-linked species-specific whole antibody from sheep (GE, NA931), and anti-rabbit IgG, HRP-linked whole antibody from donkey (GE, NA934V). All secondary antibodies were used at a 2000× dilution in 5% skim milk except for anti-mouse HRP antibody, which was used at a 5000× dilution with the anti β-actin primary antibody. Detailed western blotting conditions are described in the Supplemental Methods.

### Other statistical analysis

Comparisons of the mean circadian period, periodicity, and half-life of two groups of samples were carried out using Student’s *t*-test (bilateral, unequal variances). Error bars represent SD. Analysis of variance (ANOVA) and Tukey’s post hoc tests was used for the comparison of three or more groups using SPSS 26 (IBM).

## Additional Information

**How to cite this article**: Tamiya, H. *et al*. Rigid Cooperation of Per1 and Per2 proteins. *Sci. Rep.* **6**, 32769; doi: 10.1038/srep32769 (2016).

## Supplementary Material

Supplementary Information
