# Supplementary material for: Rigid Cooperation of Per1 and Per2 proteins
Source: Sci Rep. 2016 Sep 9;6:32769. doi: 10.1038/srep32769 (PMC5016722; doi:10.1038/srep32769)
Supplement: Supplementary Information [file srep32769-s1.pdf]

Rigid Cooperation of Per1 and Per2 proteins

Hiroyuki Tamiya[1], Sumito Ogawa[1*], Yasuyoshi Ouchi[2], Masahiro Akishita[1]

[1]Department of Geriatric Medicine, Graduate School of Medicine, The University of Tokyo, Tokyo, Japan

[2]Federation of National Public Service Personnel Mutual Aid Associations Toranomon Hospital, Tokyo, Japan

To whom correspondence should be addressed: Sumito Ogawa, MD, PhD; Department of Geriatric Medicine, Graduate School of Medicine, The University of Tokyo; 7–3–1 Hongo, Bunkyo-ku, Tokyo 113–8655, Japan, Tel: +81-3-5800-8830, Fax: +81-3-5800-6529, Email: suogawa-tky@umin.ac.jp

Supplemental Figure Legends

Supplemental Figure S1. Magnified photo of Figure 3Bc. The interface of the 12-LED–based lighting system.

Supplemental Figure S2. Representative actograms of Figure 3F (Full Span). The full span of the experiments are shown. The environmental period (the period of the light cycle) under gradually changing light is shown on the right axis of the actogram. Twenty-four h is the antiphase to the room outside of the rack to block the effects of light leak.

Supplemental Figure S3. Representative actograms of Figure 3F sorted by environmental period. Because the order of the environmental period was different between the genotypes, we sorted the actograms in the order of the environmental period.

Supplemental Figure S4.

Raw PCR and quantitative PCR data to confirm the genome structure of the Per2(WT) and Per2(FASPS)-rescued *Per2*$^{(-/-)}$ ES cells shown in Figure 5. (A) Per2(WT) and (B) Per2(FASPS) Screening and Arm PCR results targeting the structure of the *Rosa26* locus. Target 3' (4.0 kb), Target 5' (9.0 kb), WT 3' (4.5 kb), and WT 5' (8.5 kb) are shown. (C) Quantitative PCR results of the puromycin resistance gene in Per2(WT) and (D) Per2(FASPS) to show that a single copy of the transgene is expressed. The numbers shown are the clone number, which corresponds to the other figures. M = Marker, Perfect DNA Markers, 0.5–12 kbp (Novagen, 69002-3); P = positive

control (Lhx1::tRFP, P(Per1)-Per2::Luc#5, Per1#22, or WT#1); N = negative control [Tris-EDTA (TE) buffer]; 1 copy = the genome from ES cells with Lhx1::tRFP knocked into *Rosa26* locus (kindly provided by Etsuo A. Susaki), which was used in the Per2(WT) analysis. The WT#3 genome was used in the Per2(FASPS) analysis. Bar, standard deviation (SD).

Supplemental Figure S5. Bioluminescence data from *Per2*$^{(-/-)}$ ES cells rescued with Per2(WT), Per2(FASPS), Per1, or a control lacking the coding region. The bioluminescence data (oscillations not detrended) from (A) Fig 5C, (B) Fig 6B, and (C) 6E are shown.

Supplemental Figure S6. *Per2* knockout-rescue system can be used to examine the phenotypes of other *Per2* mutants. (A) Constructs used in *Per2* knockout-rescue experiment with *Per2* mutants besides FASPS. (B) Period of WT- and TrCP-rescued *Per2*$^{(-/-)}$ ES cells. Two independent TrCP-rescued ES cell lines were differentiated and analysed. Bar, standard deviation (SD). (C and D) Detrend/baseline oscillation (C) and period length (D) of Per2(mut6)-rescued *Per2*$^{(-/-)}$ ES cells. Two independent ES cell lines (N=4 samples each) were differentiated and analysed in a single experiment. mut6: mutations near the FASPS sites. Values represent mean±SD.

Supplemental Figure S7. Western blot analysis of Per1 and Per2 in Per1-rescued and Per2-rescued *Per2*$^{(-/-)}$ ES cells. (A) Per1(#16)- and Per2(#1)-rescued *Per2*$^{(-/-)}$ cells were analysed. The control cells are the same cells used as the controls in the half-life analysis (Figure S8C) seven days after transfection. (B) The same analysis shown in (A), but with NIH3T3 cells three days

after transfection as the control. A mock (pMU2 vector) control was also tested. Tx = transfection. Both ES cells were Per2 null and expressed similar levels of Per1. The expression of Per1::Luc and Per2::Luc was detected only in the rescued ES cells.

We could not determine the ratio of Per1 and Per2 because the Per1 and Per2 antibodies are different, and the transfer efficiency of the native protein and luciferase-fusion protein from the acrylamide gel to the membrane varied due to molecular weights that differed by 60 kD. Hence, we can only say that Per1::Luc and Per2::Luc were expressed. Moreover, we could not detect the Flag-tag with the M2 antibody. We observed the bands only with anti-mouse IgG antibody (A) and without transfection of Flag-Per1 or Flag-Per2 (B). Taken together, all bands appear to be non-specific bands. However, it would be difficult to detect a 1XFlag-tagged protein compared to a 3XFlag-tagged protein in a relatively low expression system compared to other systems like HEK293 cells.

Supplemental Figure S8. Half-life analysis using luminescence and western blotting. (A) The results of six independent half-life analysis experiments are shown. Statistically significant differences were likewise detected (*t*-test: Per2 (N=13) vs Per1 (N=14), P=0.003; Mann-Whitney U-test (Wilcoxon rank sum test): Per2 (N=13) vs Per1 (N=14), P<0.001; Paired *t*-test using the mean values from each experiment, Per2 (N=6) vs Per1 (N=6), P=0.002). A boxplot of all half-lives is also shown (right). (B) Half-life analyses with dispersed phase conditions are shown. Three independent experiments were mixed and analysed. Clearer results are seen. Western blot analysis (C) and half-life analysis (D) of Per1 and Per2 using cycloheximide (CHX). NT = no treatment. Relative band intensity was calculated using Image J. Exponential regression lines were calculated

and are likewise indicated. The time 0 value represents the mean of NT and time 0. Per1 appeared more stable in this experiment [P=0.01: Regression analysis based on the null hypothesis that the slope of log (Per1/Per2)/time = 0].

Supplemental Figure S9. Period length and protein half-life are not always correlated. A) Mean half-life in NIH3T3 cells plotted against mean length of circadian period in rescued *Per2* knockout ES cells. Values represent mean±standard deviation (SD). B) Our hypothesis on the molecular mechanism underlying association with period length and cooperativity of Per1 and Per2. Ub: ubiquitin; CK1, casein kinase 1.

Supplemental Table S1 Circadian period length under constant light conditions for 14 days using Brd mutant mice.

	WT (N=12)	*Per2*$^{(+/-)}$ (N=14)	*Per2*$^{(-/-)}$ (N=5)
LL (hours)[1]	25.4±0.5[2]	24.1±0.4	24.1±0.1
DD (hours)[1]	24.2±0.2	24.1±0.5	23.0±0.4

[1]The same protocol detailed in Figure 1 was used; [2]mean±SD

Supplemental Table S2. All of the F and P-values from the Tukey's tests shown in Figure 1.

DD: F= 82.164<br>P<0.001	*Per1(-/-)*	*Per1(+/-)*	*WT*	*Per2 (+/-)*	*Per2 (-/-)*
Per1(-/-)	-	P=0.035	P<0.001	P=0.035	P<0.001
Per1(+/-)		-	P=0.513	P=1.000	P<0.001
WT			-	P=0.513	P<0.001
Per2 (+/-)				-	P<0.001
Per2 (-/-)					-

LL: F= 99.287<br>P<0.001	*Per1(-/-)*	*Per1(+/-)*	*WT*	*Per2 (+/-)*	*Per2 (-/-)*
Per1(-/-)	-	P=0.001	P<0.001	P<0.001	P<0.001
Per1(+/-)		-	P=0.001	P<0.001	P<0.001
WT			-	P=0.282	P<0.001
Per2 (+/-)				-	P<0.001
Per2 (-/-)					-

Supplemental Table S3. All of the F and P-values from the Tukey's tests shown in Figure 2.

	F	*WT vs Per1(-/-)*	*WT vs Per2(-/-)*	*Per1(-/-) vs Per2(-/-)*
DD	2.020 (P=0.149)	P=0.174	P=0.977	P=0.248
LL	10.344 (P<0.001)	P=0.001	P=0.001	P=0.998

Supplemental Table S4. All of the F and P-values from the Tukey's tests shown in Figure 3.

2 weeks	*F*	*WT vs Per1(-/-)*	*WT vs Per2(-/-)*	*Per1(-/-) vs Per2(-/-)*
22 h	21.835 (P<0.001)	P=0.054	P<0.001	P=0.003
23 h	11.263 (P<0.001)	P=0.001	P=0.038	P=0.150
24 h	5.223 (P=0.019)	P=0.015	P=0.197	P=0.363
25 h	10.874 (P=0.001)	P=0.002	P=0.923	P=0.004
26 h	65.476 (P<0.001)	P<0.001	P<0.001	P<0.001
27 h	415.352 (P=0.001)	P<0.001	P<0.001	P<0.001

1 week	*F*	*WT vs Per1(-/-)*	*WT vs Per2(-/-)*	*Per1(-/-) vs Per2(-/-)*
22 h	18.012 (P<0.001)	P=0.012	P<0.001	P=0.037
23 h	4.542 (P<0.001)	P=0.049	P=0.049	P=1.000
24 h	0.846 (P=0.449)	P=0.441	P=0.943	P=0.632
25 h	0.728 (P=0.499)	P=0.477	P=0.910	P=0.725
26 h	36.605 (P<0.001)	P=0.003	P=0.001	P<0.001
27 h	100.662 (P<0.001)	P<0.001	P<0.001	P<0.001

Supplemental Table S5. All of the F and P-values from the Tukey's tests shown in Figure 7A.

F = 29.414, P<0.001	Per2(WT)	Per2(FASPS)	no coding	Per1	Per2(TRCP)	Per2(mut6)
Per2(WT)	-	P<0.001	P=0.003	P=0.01	P=1.000	P<0.001
Per2(FASPS)		-	P=0.27	P<0.001	P<0.001	P=0.996
no coding			-	P=0.556	P=0.01	P=0.115
Per1				-	P=0.08	P<0.001
Per2(TRCP)					-	P<0.001
Per2(mut6)						-

Supplemental Methods

Recombinant Plasmid Production – We have used the format "pXXX" to describe the plasmid backbone and "P(XXX)" to describe promoters. With the *Per2* promoter, we refer to the distal (3518 bps) promoter as the *Per2* long promoter (P(Per2L)) and the proximal (296 bps) promoter as the *Per2* short promoter (P(Per2s)). Reporter analysis showed that these two promoters have the same circadian phase of promoter oscillation.[1] "Luc(c)" refers to the luciferase gene inserted into the PI-Psp1 site and positioned to fuse with the coding region for the C-terminal end of the gene of interest. "XXX::Luc" describes the C-terminal fusion protein encoded by the XXX and luciferase genes.

The pCMV-Sport2 mPer1 plasmid was kindly provided by Cheng Lee (Baylor College of Medicine) through AddGene.[2] The pTVCI2-DT-RareCutSite-ROSA 4 kb arm-Gateway<attR1/2>-FRT<P(PGK)–puro–polyA>–ROSA short arm 4 kb plasmid, and the pCR8-P(Per2L)-Luc(c) plasmid were kindly provided by Maki Ukai-Tadenuma (RIKEN). The pMU2-Per2, pMU2,[3] pCAGGS-ROSA-TALEN-N153C63-R, and pCAGGS-ROSA- TALEN- N153C63-L plasmids were kindly provided by Hideki Ukai (RIKEN).

pMU2-Luc(c) was constructed as follows. Luc(c) was obtained by digestion of pCR8-P(Per2L)-Luc(c) with PI-Psp1 and PI-Sce1 (both New England Biolabs), isolated by agarose gel electrophoresis, extracted from the gel, and cloned into pMU2 to generate pMU2-Luc(c).

FASPS, mut6, β-TrCP binding site mutants of *Per2* were generated from pMU2-Per2 by inverse PCR using KOD Plus Neo (Takara) according to the manufacturer’s protocol. PCR was carried out under conditions of 35 cycles of 98°C for 10 s and 68°C for 1 min 56 s; followed by 68°C for

3 min. The PCR products were separated by agarose gel electrophoresis, isolated from the gel, phosphorylated with T4 polynucleotide kinase (Takara), and self-ligated using the DNA Ligation Kit, Mighty Mix (Takara). Primers for each mutant are as follows. FASPS: (forward) GGTGTGGTGTCCCTCACCAGCCAGT and (reverse) CTCGGCCTTGCCTGGCAGCGT; mut6: (forward) CGCCTACGCCGCCGCCATCGTGCATGTGGGCGACAAAAAG and (reverse) CACTGGGCGGTGAGGGCCACCACACTCTCGGCCTTGCCT; TrCP: (forward) GCCTATGGGAGCCTGGGCAGTAACG and (reverse) ACTGGCGCCGCTGTGGGGGACAGGCT.

Plasmid pMU2-Per1::Luc was constructed as follows. First, the *mPer1* coding region was amplified using pCMV-Sport2 mPer1 as template and primers I-Sce1_mPer1_F_modified: ATTACCCTGTTATCCCTAATAGTGGTCCCCTAGAAGGGGC and PI-Psp1_mPer1_R: ACCCATAATACCCATAATAGCTGTTTGCCAGCTGGTGCTGTTTTCTTCTG using KOD Plus Neo according to the manufacturer's protocol. PCR was carried out under conditions of 98°C for 2 min; followed by 35 cycles of 98°C for 10 s and 68°C for 1 min 56 s; followed by 68°C for 3 min. PCR products were separated by agarose gel electrophoresis, extracted from the gel, digested with PI-Psp1 and I-Sce1 (New England Biolabs), and cloned into pMU2-Luc(c).

To construct pMU2-Per2(WT)::Luc, pMU2-Per2(FASPS)::Luc, pMU2-Per2(mut6)::Luc, and pMU2-Per2(TrCP)::Luc, coding regions were obtained from pMU2-Per2(WT), pMU2-Per2(FASPS), pMU2-Per2(mut6), and pMU2-Per2(TrCP), respectively, by digestion with PI-Psp1 and I-Sce1, and cloned into vector pMU2-Luc(c).

Vectors pTVCI2-P(Per2L)-Per2(or Per1)::Luc and pTVCI2-P(Per2L)-Luc were constructed as follows. pMU2-Per2 (WT or mutants) and pMU2-Per1::Luc were digested with PI-Psp1, I-Sce1

(both New England Biolabs) and Ssp1 (Takara), fragments were separated by agarose gel electrophoresis, isolated from the gel, and cloned into pCR8-P(Per2L)-Luc(c) to generate pCR8-P(Per2L)- Per2(or Per1)::Luc(c). These vectors were recombined with pTVCI2-DT-RareCutSite-ROSA 4 kb arm-Gateway<attR1/2>-FRT<P(PGK)–puro–polyA>–ROSA short arm 4 kb, using LR clonase (Invitrogen) to generate pTVCI2-P(Per2L)-Per2(Per1, or null)::Luc. Coding regions of all plasmids used in this study were validated by full sequencing.

Animals and Behavioural Analysis – All animal experiments were performed in the Center for Developmental Biology and were approved by the Animal Care Committee of the Center for Developmental Biology (approval ID: AH15-10-22)) and all experimental procedures were performed according to the guidelines of the Animal Care Committee of Center for Developmental Biology. Animal behaviour analyses were performed using a behavioural analysis rack (Nihon-Ika) as previously described.[4] These racks are a special order product based on Clean Rack (Nihon-Ika, CR-1600S) and are made specifically for exposing mice to light-dark conditions. Briefly, locomotor data were collected using an infrared sensor (NS-AS01, Neuroscience), plotted using ClockLab software (Actimetrix) and the double-plot method,[5] and circadian period length was calculated using a chi-square periodogram.[6] All mice used in the behavioural analysis were male. Mice of the same genotype used in different experiments were of different ages and were generated from different parental genotypes obtained from different sources. Therefore, generation of mice is described by experiment.

To generate mice of genotypes shown in Figure 1, we used *Per1* and *Per2* knockout C57B6/J mice of the ldc line.[7] $Per1^{(-/-)}Per2^{(-/-)}$ males (kindly provided by Genshiro A. Sunagawa of RIKEN) were

mated with WT *Per1*$^{(+/+)}$*Per2*$^{(+/+)}$ C57B6/J females (Japan SLC) to generate F1 *Per1*$^{(+/-)}$*Per2*$^{(+/-)}$ mice for balancing genetic backgrounds. Next, we mated the F1 mice, obtained 97 male F2 mice, and performed genotyping. We used six mice from each of the following genotypes: *Per1*$^{(+/+)}$*Per2*$^{(+/+)}$, *Per1*$^{(+/-)}$*Per2*$^{(+/+)}$, *Per1*$^{(-/-)}$*Per2*$^{(+/+)}$, *Per1*$^{(+/+)}$*Per2*$^{(+/-)}$, and *Per1*$^{(+/+)}$*Per2*$^{(-/-)}$. Because we obtained only four *Per1*$^{(+/+)}$*Per2*$^{(+/+)}$ mice and two *Per1*$^{(+/+)}$*Per2*$^{(-/-)}$, we used six more WT mice (Japan SLC) and six more *Per1*$^{(+/+)}$*Per2*$^{(-/-)}$ mice from the mating of *Per1*$^{(+/-)}$*Per2*$^{(-/-)}$. At first, we analysed mice from the mating of *Per1*$^{(+/-)}$*Per2*$^{(+/-)}$ and other mice separately. However, the results were similar and finally we analysed all mice together. Mice were 8–16 weeks of age at the start of the experiments (Figure 1).

To generate mice of genotypes shown in Figure 2, we mated *Per1*$^{(-/-)}$*Per2*$^{(+/+)}$ males and females and *Per1*$^{(+/+)}$*Per2*$^{(-/-)}$ males and females described above and obtained *Per1*$^{(-/-)}$ and *Per2*$^{(-/-)}$ mice. WT C57B6/J mice were purchased from Charles River Laboratories. Only male mice, 16–24 weeks of age at the start of the experiments, were used. The experiments were conducted using a crossover design. Periodicity is defined as the mean amplitude (Q[p]) minus the level of significance at the determined period length within ±1 h of the circadian period length, determined using the chi-square periodogram. We calculated the periodicity for weeks 3 and 4 after initiation of constant light conditions because most of the WT mice became arrhythmic approximately two weeks after initiation of constant light conditions.

To generate mice of genotypes shown in Figure 3, we used *Per1*$^{(-/-)}$ and *Per2*$^{(-/-)}$ mice from the lines obtained in the experiment shown in Figure 1. WT C57B6/J mice were from Japan SLC. All mice used in the experiment were more than 8 weeks old.

LED-based lighting system – An LED-based lighting system containing 12 lights was developed. This system uses the proportionate relationship between light strength and electric current. Linear lighting power (256 levels) was produced using a combination of eight resistance units. The main unit measured 160 mm × 210 mm × 90 mm and contained six cords each on the front and back. Each cord regulated one LED disk, and one disk consisted of nine LEDs (Biotex). The six front and six back LED disks can be differentially regulated. The interface was regulated on a personal computer to generate levels 0–255 of light intensity. The system included three modes. In test mode, light level can be set at any intensity; in time command mode, light can be stepped-up at any interval, to any level, and for any duration; and in file data mode, the text file of any pattern of light intensity can be read. In file data mode, 12 mice can be exposed to any pattern of light intensity, with 256 levels at 1-min resolution to 12 mice, e.g., 6 mice exposed to two patterns each. Text files spanning more than 10 years can be read in this system. LED disks are set beside the ClockLab infrared sensor. For the experiments, mice were housed one/cage. The lighting program was used with the following formula: I=128+[0.5+$A sin 2\pi(x_{min}/(T_h*60))$], where I represents light intensity level (integer from 0–255); A represents amplitude of light intensity level (we used 127); T_h represents period of environmental light cycles [we performed the experiment using periods of 22–27 h, in increments of 1 h, (but not in that order) for 2 weeks each]; and x_{min} represents the number of minutes after the start of the experiment. For the chi-square periodogram, more than 10 days are desired to avoid short-term random variations.[8] However, the transient or aftereffects were quite large in the first week because we could not connect the phase of each environmental cycle in these experiments. Thus, we performed both chi-square periodograms for a duration of 2 weeks and one with a final week for each environmental period. Food was put in a feeder basket

No. 4 (CLEA Japan), not on the top of the cage, in order not to come between the light source and the mouse. To achieve rigorous light protection, we used a light protection curtain with a protection rate of 99.9% (Sangetsu) on the behavioural analysis rack.

Light intensity levels of 0–255 on the floor of the rack just below the LED were monitored using a CL-200A Chroma Meter (Konica Minolta) and changed almost linearly. Resistance of the main unit can be increased to 10× and 100×, i.e., 1/10 and 1/100 of the electric current and lighting strength, respectively. To avoid the mice sensing constant light conditions,[9] we chose 256 levels ranging from approximately 0.01 to 5.4 lux.

The light control program was carried out using Visual Basic (Microsoft) and the following code:

```
Option Explicit
Dim DoNothing As Boolean
Dim n As Integer
Dim LightValue(2) As Integer
Dim Interval As Integer
Dim StepData As Integer
Dim MaximumValue As Integer
Dim Flag As Integer
Dim strFileName1 As String
Dim strFileName2 As String
Dim CountFlag As Integer

Private Declare Function OpenDevice Lib "k8055d.dll" (ByVal CardAddress As Long) As Long
```

```
Private Declare Sub CloseDevice Lib "k8055d.dll" ()
Private Declare Function ReadAnalogChannel Lib "k8055d.dll" (ByVal Channel As Long) As Long
Private Declare Sub ReadAllAnalog Lib "k8055d.dll" (Data1 As Long, Data2 As Long)
Private Declare Sub OutputAnalogChannel Lib "k8055d.dll" (ByVal Channel As Long, ByVal Data As Long)
Private Declare Sub OutputAllAnalog Lib "k8055d.dll" (ByVal Data1 As Long, ByVal Data2 As Long)
Private Declare Sub ClearAnalogChannel Lib "k8055d.dll" (ByVal Channel As Long)
Private Declare Sub SetAllAnalog Lib "k8055d.dll" ()
Private Declare Sub ClearAllAnalog Lib "k8055d.dll" ()
Private Declare Sub SetAnalogChannel Lib "k8055d.dll" (ByVal Channel As Long)
Private Declare Sub WriteAllDigital Lib "k8055d.dll" (ByVal Data As Long)
Private Declare Sub ClearDigitalChannel Lib "k8055d.dll" (ByVal Channel As Long)
Private Declare Sub ClearAllDigital Lib "k8055d.dll" ()
Private Declare Sub SetDigitalChannel Lib "k8055d.dll" (ByVal Channel As Long)
Private Declare Sub SetAllDigital Lib "k8055d.dll" ()
Private Declare Function ReadDigitalChannel Lib "k8055d.dll" (ByVal Channel As Long) As Boolean
Private Declare Function ReadAllDigital Lib "k8055d.dll" () As Long
Private Declare Function ReadCounter Lib "k8055d.dll" (ByVal CounterNr As Long) As Long
Private Declare Sub ResetCounter Lib "k8055d.dll" (ByVal CounterNr As Long)
```

```
Private Declare Sub SetCounterDebounceTime Lib "k8055d.dll" (ByVal CounterNr As Long, ByVal DebounceTime As Long)

Private Sub Check3_Click(Index As Integer)
    Dim i As Long
    Dim n As Long
    n = 0
    For i = 0 To 7
    '       n = n + Check3(i).Value * (2 ^ i)
    Next
    If Not DoNothing Then WriteAllDigital n
End Sub

Private Sub Check4_Click()


End Sub

Private Sub ClearAllAnalog1_Click()
    ClearAllAnalog
    LightValue(0) = 0
    LightValue(0) = 0
    LightValue(1) = 0
```

```
        Label3.Caption = LightValue(0)
        OutputAnalogChannel 1, LightValue(0)
        Label10.Caption = LightValue(1)
        OutputAnalogChannel 2, LightValue(1)

        'VScroll2.Value = 0
        'Label3.Caption = 0
        'Label4.Caption = 0
End Sub


Private Sub Combo1_Click(Index As Integer)
        If Index = 0 Then
                Interval = Val(Combo1(0).Text)
        ElseIf Index = 1 Then
                Interval = Val(Combo1(1).Text)
        End If
End Sub

Private Sub Combo2_Click()
        Select Case Combo2.Text
                Case "TEST"
```

```
            Frame2.Enabled = True
            Frame3.Enabled = False
            Frame4.Enabled = False
            Label14.Enabled = False
            Start.Enabled = False
            Command2.Enabled = False
        Case "TIME COMMAND"
            Frame2.Enabled = False
            Frame3.Enabled = True
            Frame4.Enabled = False
            Label14.Enabled = False
            Start.Enabled = True
            Command2.Enabled = False
        Case "FILE DATA"
            Frame2.Enabled = False
            Frame3.Enabled = False
            Frame4.Enabled = True
            Label14.Enabled = True
            Start.Enabled = False
            Command2.Enabled = False
    End Select
    Timer1.Enabled = False
```

```
        'LightValue(0) = 0
        'Label3.Caption = LightValue(0)
        'OutputAnalogChannel 1, LightValue(0)
End Sub

Private Sub Command1_Click()
        CommonDialog1.ShowOpen
        strFileName1 = CommonDialog1.FileName
        Command2.Enabled = True

End Sub

Private Sub Command2_Click()
Dim STime As String
Dim Loct As Integer
        Command2.Enabled = False
        If Text3.Text = "00:00" Then
                Timer1.Enabled = True
                Close #1
                Close #2
                Open strFileName1 For Input As #1
                Open strFileName2 For Input As #2
```

```
        Command3.Enabled = True
    Else
        Do Until Text3.Text = STime And Text5.Text = Date
            Loct = InStr(Time, ":")
            Loct = InStr(Loct + 1, Time, ":")
            STime = Left$(Time, Loct - 1)
            DoEvents
        Loop
        Timer1.Enabled = True
        Close #1
        Close #2
        Open strFileName1 For Input As #1
        Open strFileName2 For Input As #2
        Command3.Enabled = True
    End If
End Sub

Private Sub Command3_Click()
    Timer1.Enabled = False
    Command2.Enabled = True
    'LightValue(0) = 0
    'Label3.Caption = LightValue(0)
```

```
    'OutputAnalogChannel 1, LightValue(0)
    'OutputAnalogChannel 2, LightValue(1)
    Command3.Enabled = False
End Sub

Private Sub Command4_Click()
    CommonDialog1.ShowOpen
    strFileName2 = CommonDialog1.FileName
    Command2.Enabled = True

End Sub

Private Sub Connect_Click()
    Dim CardAddress As Long
    Dim h As Long
    CardAddress = 0
    CardAddress = 3 - (Check1(0).Value + Check1(1).Value * 2)
    h = OpenDevice(CardAddress)
    Select Case h
        Case 0, 1, 2, 3
            Label1.Caption = "Card " + Str(h) + " connected"
        Case -1
```

```
            Label1.Caption = "Card " + Str(CardAddress) + " not found"
    End Select
    'If h >= 0 Then Timer1.Enabled = True
End Sub

Private Sub Form_Initialize()
    DoNothing = False
    n = 7
    Combo1(0).AddItem 1
    Combo1(0).AddItem 2
    Combo1(0).AddItem 5
    Combo1(0).AddItem 10
    Combo1(0).AddItem 30
    Combo1(0).AddItem 60
    Combo1(0).Text = 1
    Interval = 1
    StepData = 1
    MaximumValue = 255
    Flag = 0

    Combo1(1).AddItem 1
    Combo1(1).AddItem 2
```

```
Combo1(1).AddItem 5
Combo1(1).AddItem 10
Combo1(1).AddItem 30
Combo1(1).AddItem 60
Combo1(1).Text = 1
Interval = 1

Combo2.AddItem "TEST"
Combo2.AddItem "TIME COMMAND"
Combo2.AddItem "FILE DATA"
Combo2.Text = "TEST"
Frame2.Enabled = True
Frame3.Enabled = False
Frame4.Enabled = False

Start.Enabled = False
Stopp.Enabled = False
Command2.Enabled = False
Command3.Enabled = False

Connect_Click
ClearAllAnalog1_Click
```

```
    Text5.Text = Date
End Sub

Private Sub Form_Terminate()
    CloseDevice
End Sub

Private Sub Option1_Click(Index As Integer)
Dim t1 As Long
    Select Case Index
    Case 0
        t1 = 0
    Case 1
        t1 = 2
    Case 2
        t1 = 10
    Case 3
        t1 = 1000
    End Select
    SetCounterDebounceTime 1, t1
End Sub
```

```
Private Sub Option2_Click(Index As Integer)
Dim t2 As Long
    Select Case Index
    Case 0
        t2 = 0
    Case 1
        t2 = 2
    Case 2
        t2 = 10
    Case 3
        t2 = 1000
    End Select
    SetCounterDebounceTime 2, t2
End Sub

Private Sub Reset1_Click()
    ResetCounter 1
End Sub

Private Sub Reset2_Click()
    ResetCounter 2
```

```
End Sub

Private Sub SetAllAnalog1_Click()
    SetAllAnalog
    LightValue(0) = 255
    'VScroll2.Value = 255
    Label3.Caption = 255
    'Label4.Caption = 255
End Sub

Private Sub SetAllDig_Click()
    Dim i As Long
    DoNothing = True
    For i = 0 To 7
    '    Check3(i).Value = 1
    Next
    DoNothing = False
    SetAllDigital
End Sub

Private Sub ClearAllDig_Click()
    Dim i As Long
```

```
    DoNothing = True
    For i = 0 To 7
'       Check3(i).Value = 0
    Next
    DoNothing = False
    ClearAllDigital
End Sub

Private Sub Start_Click()
    Timer1.Enabled = True
    Start.Enabled = False
    Stopp.Enabled = True
    CountFlag = 1
End Sub

Private Sub Stopp_Click()
    Timer1.Enabled = False
    Stopp.Enabled = False
    Start.Enabled = True
End Sub

Private Sub Text2_Change()
```

```
    MaximumValue = Val(Text2.Text)
End Sub


Private Sub Text4_Change()
    StepData = Val(Text4.Text)
End Sub

Private Sub Timer1_Timer()
    Select Case Combo2.Text
    Case "TIME COMMAND"
        If Timer Mod Interval * 60 = 0 Then
        'If Int(Timer) Mod Interval = 0 Then
            If Flag = 0 Then
            Flag = 1
                If CountFlag = 1 Then
                    LightValue(0) = LightValue(0) + StepData
                    LightValue(1) = LightValue(1) + StepData
                ElseIf CountFlag = 0 Then
                    LightValue(0) = LightValue(0) - StepData
                    LightValue(1) = LightValue(1) - StepData
                End If
```

```
                If LightValue(0) > MaximumValue Then CountFlag = 0
                If LightValue(0) < 0 Then CountFlag = 1
                If LightValue(1) > MaximumValue Then CountFlag = 0
                If LightValue(1) < 0 Then CountFlag = 1
            End If
        Else
            Flag = 0
        End If
        If LightValue(0) > 255 Then LightValue(0) = 255
        If LightValue(0) < 0 Then LightValue(0) = 0
        If LightValue(1) > 255 Then LightValue(1) = 255
        If LightValue(1) < 0 Then LightValue(1) = 0
        Label3.Caption = LightValue(0)
        OutputAnalogChannel 1, LightValue(0)
        Label10.Caption = LightValue(1)
        OutputAnalogChannel 2, LightValue(1)
    Case "FILE DATA"
'        Timer1.Enabled = True
        If Timer Mod Interval * 60 = 0 Then
        'If Int(Timer) Mod Interval = 0 Then
            If Flag = 0 Then
                Input #1, LightValue(0)
```

```
            Input #2, LightValue(1)
            Flag = 1
            If LightValue(0) > MaximumValue Then LightValue(0) = 0
            If LightValue(0) > 255 Then LightValue(0) = 0
            If LightValue(1) > MaximumValue Then LightValue(0) = 0
            If LightValue(1) > 255 Then LightValue(0) = 0
        End If
Else
        Flag = 0
            If EOF(1) = True Then
                Close #1
                Open strFileName1 For Input As #1
                Input #1, LightValue(0)
            End If
            If EOF(2) = True Then
                Close #2
                Open strFileName2 For Input As #2
                Input #2, LightValue(1)
            End If
End If
Label3.Caption = LightValue(0)
OutputAnalogChannel 1, LightValue(0)
```

```
            Label10.Caption = LightValue(1)
            OutputAnalogChannel 2, LightValue(1)
'           Timer1.Enabled = True
        End Select
        VScroll1.Value = LightValue(0)
        VScroll2.Value = LightValue(1)

End Sub

Private Sub Timer2_Timer()
        ClearDigitalChannel n + 1
        'Check3(n).Value = 0
        n = n + 1
        If n = 8 Then n = 0
        SetDigitalChannel n + 1
        'Check3(n).Value = 1
End Sub

Private Sub VScroll1_Scroll()
        LightValue(0) = VScroll1.Value
        Label3.Caption = LightValue(0)
        OutputAnalogChannel 1, LightValue(0)
```

```
End Sub

Private Sub VScroll2_Scroll()
    LightValue(1) = VScroll2.Value
    Label10.Caption = LightValue(1)
    OutputAnalogChannel 2, VScroll2.Value
End Sub
```

Genotyping – Genotyping of *Per1* and *Per2* knockout mice was performed as previously described[7] with the following modification. DNA extraction was carried out using sodium hydroxide (NaOH)-based methods, and PCR was carried out using Mighty Amp DNA polymerase ver. 2 (Takara R071A), 0.3 μM forward primer, 0.15 μM reverse primer, and 0.15 μM Neo primer under conditions of 98°C for 2 min; followed by 35 cycles of 98°C for 10 s, 64.8°C for 15 s, and 68°C for 60 s; and 68°C for 2 min. The protocols for amplification of *Per1* and *Per2* differed only in the primers used.

ES cells and culture – Per2::Luciferase KI/KI (*Per2::Luc KI/KI*)[10] ES cells were kindly provided by Etsuo A. Susaki (University of Tokyo). *Per2*$^{(-/-)}$ ES cells were established by Naoshi Koide (RIKEN) from mice generated in this study. Behavioural analysis showed that chimeric mice generated from *Per2*$^{(-/-)}$ ES cells cultured in the presence of three inhibitors (3i; SU5402 for FGFR, PD184352 for ERK, and CHIR99021 for GSK3) exhibited the same circadian period length under conditions of constant light (light-light, LL) and constant dark (dark-dark, DD) as *Per2*$^{(-/-)}$ mice

generated from mating.[11]

ES cells were cultured in the absence of feeder cells in 60-mm dishes (Falcon 353004) that had been coated with 0.1% autoclaved porcine skin gelatine (Sigma G2500) for at least 30 min at 37°C. Cells were cultured in ES medium consisting of Glasgow Minimum Essential Medium (GMEM, Gibco 11710-035), 10% KnockOut™ Serum Replacement (KSR; Gibco 10828-028), 1% foetal bovine serum (FBS) (JRH Bioscience 12603C-500ML; lot: 4C0702, 6K0357 or Sigma 17012-500ML; lot: S.12H183, heat inactivated), 1 mM sodium pyruvate (Gibco 11360-070), 1× MEM Non-Essential Amino Acids (Gibco 11140-050), 100 μM β-mercaptoethanol (Wako, β-ME) [12]. ES medium was filtered through 0.22-μm SteriCup filter (Millipore SCGPU05RE). Then, two inhibitors (2i), 3 μM CHIR99021 (Axon 1386, 1000× stock in DMSO) and 1 μM PD0325901 (Wako 162-25291, 1000× stock in DMSO)[13] were added and medium was filtered as before. ESGRO mouse leukaemia inhibitory factor (mLIF, Millipore ESG1106) was added to a final concentration of 2×10^3 U/mL.[14] Cells were passed every other day onto 60-mm culture dishes. Cells were first washed with Dulbecco's Phosphate-Buffered Saline (DPBS, without calcium and magnesium, Gibco 14190-144) and incubated in 0.5 mL 0.05% trypsin/0.48 mM EDTA (Gibco 25300-054) at 37°C for 2 min followed by addition of 1.0 mL ES medium 2i(-)LIF(-) and pipetting up and down with a blue tip 10 times. ES medium 2i(-)LIF(-) (3.0 mL) was then added, and cells were pipetted with a 5-mL pipet and passed through a 35-μm Cell Strainer (BD Falcon 352235). After centrifugation at 1000 rpm for 5 min at room temperature in a bucket centrifuge (Tomy LC-121, EX-125, EX-126), supernatant was removed, 1.0 mL ES medium 2i(-)LIF(-) was added, and cells were pipetted with a blue tip 20 times. Cells (2×10^5) were plated in 4 mL ES medium 2i(+)LIF(+) on gelatine-coated 60-mm dishes and incubated at 37°C in a humidified atmosphere

of 5% CO_2.

Gene targeting and colony picking – We used *Per2*$^{(-/-)}$ ES cells for the *Per2* knockout-rescue experiments for three primary reasons. First, we could confirm the knockout ES cells by checking the free run period of chimeric mice generated using the knockout ES cells. In the cellular circadian experiments, it is very important to determine whether the oscillation can be rescued in the established cell line because the circadian system could be lost during the establishment of the cell line. Second, we could not confirm the rescue condition with 1) transient transfection of *Per1/2* double knockout and *Per2* knockout mouse embryonic fibroblasts, 2) gene targeting with *Per1/2* double knockout ES, or 3) transient transfection of *Per1/2* double knockout and *Per2* knockout ES cells. Third, we could easily apply the same system as the gene targeting protocols used for the mouse genetics experiments. On day 0, *Per2*$^{(-/-)}$ ES cells (5×10^5) were plated in 2.0 mL ES medium 2i(+)LIF(+) on 35-mm culture dishes (Falcon 353001) that had been coated with 0.2% gelatine. Five hours after plating, cells were transfected with 1 μg targeting vector, 2 μg pCAGGS-ROSA-TALEN-N153C63-R, and 2 μg pCAGGS- ROSA- TALEN-N153C63-L using Xfect Stem mESC reagent (Clontech) according to the manufacturer's protocol with the following modifications. Immediately prior to addition of transfection reagents, 1.0 mL of culture medium was removed from each dish. Ten minutes after addition of transfection reagents, 5% bovine serum albumin (BSA, Sigma) that had been filtered with a Millex GP 0.22-μm filter (Millipore, SLGP033RS) was added to a final concentration of 1% to diminish cell damage. Medium was changed 4 h after transfection and then once daily. On day 3, cells were harvested and seeded at 1×10^6 on 0.2% gelatine-coated 60-mm dishes. Selection with 1.2 μg/mL puromycin (Sigma, P8833-10MG) was

carried out for 24 h on days 4 and 6. ES clones were picked on or after day 8 as follows. The phase contrast microscope was placed in the laminar flow hood, and cells were washed twice with DPBS with calcium and magnesium (Gibco 14040-141). DPBS with calcium and magnesium (3 mL) was added to each dish, and then single round, smooth-edged colonies were picked up with 10 μL medium using an autoclaved yellow tip and treated with 50 μL 0.05% trypsin/0.48 mM EDTA (Gibco 25300-054) in autoclaved 1.5 mL microtubes (Watson, WTS-131415C) for 5 min at 37°C. ES medium 2i(-)LIF(-) (100 μL) was added to tubes, and cells were pipetted with a yellow tip. Cells were plated in 500 μL ES medium 2i(+)LIF(+) on 0.2% gelatine-coated 24-well plates (Techno Plastic Products). Approximately one third of the cells were lysed and used in PCR screening.

PCR Screening – Pellets of picked ES cell colonies were washed with DPBS without calcium and magnesium and treated with 50 μL 0.2 mg/mL proteinase K (PCR grade, Roche) and incubated at 55°C for 30 min and then 95°C for 10 min in a Thermal Cycler Dice (Takara). After centrifugation at 2500 rpm for 10 min using a plate centrifuge (Plate Spin, Kubota), 5 μL supernatant was used in PCR to amplify the 3' arm of the targeted allele (4.0 kb) using KOD Fx Neo (Toyobo) with 0.3 μM of each primer in 25 μL. Primer sequences were as follows:

targeted allele 3' arm forward: TCCATCAGAAGCTGGTCGATC,

targeted allele 3' arm reverse: GGTGAAATGCTTGACTCCTAGACTT. Reactions were carried out under conditions of 98°C for 2 min; followed by 30 cycles of 98°C for 10 s, 60°C for 30 s, and 68°C for 4 min. Reaction products were separated by Tris-acetate-EDTA (TAE) 0.8% agarose gel electrophoresis with Perfect DNA Markers, 0.5–12 kbp (Novagen). A 0.8% (w/v) agarose

(Agarose S, Nippon Gene) Tris-acetate-EDTA (TAE) 0.5 μg/mL ethidium bromide gel was used. The Mupid-2plus system (Mupid) was used for electrophoresis and the gels were visualised using a FAS 3 transilluminator (NIPPON Genetics).

Arm PCR – The integrity of the genome of targeted ES cells was confirmed by arm PCR instead of southern blotting. Confluent ES clones in 60-mm dishes were harvested and lysed in 500 μL autoclaved sarcosine lysis buffer (0.5% N-lauroylsarcosine (Wako), 10 mM Tris (pH 7.5), 10 mM NaCl, 10 mM EDTA) and 50 μL 10 mg/mL proteinase K at 65°C for 1 h with occasional gentle inversion. One microliter of 10 mg/mL bovine pancreas RNase A (Roche), which had been DNase-inactivated at 80°C for 30 min, was added to the lysates, followed by incubation for 15 min at room temperature. DNA was then purified with phenol, phenol/chloroform/isoamyl alcohol (both Nacalai), and chloroform (Wako), precipitated with 3 M sodium acetate (NaOAc, Thermo Scientific), pH 5.2, and isopropanol (Wako), washed with 70% ethanol (Wako), and resuspended in Tris-EDTA (TE), pH 7.5. DNA concentration was determined using a NanoDrop 1000 spectrophotometer (Thermo Scientific), and integrity was confirmed by 0.8% TAE agarose gel electrophoresis. To confirm homologous recombination of targeting vector to correct region of the genome, three PCRs were carried out: target 5' (9.0 kb), WT 5' (8.5 kb), and WT 3' (4.5 kb) using KOD Fx Neo (Toyobo) according to manufacturer's protocol with 0.3 μM each primer and 200 ng DNA/25 μL reaction mixture. Primer sequences were as follows:

Targeted allele 5' arm forward: GACAGGGTCTCTTACTAGCCTAAAC,

Targeted allele 5' arm reverse: GTACCAGAAGGGCGAATTCGGAGCC,

WT 5' arm forward: TGCTGGCCTACTGCTGCCTCGATCTTAC,

WT 5' arm reverse: AGGACAACGCCCACACACCAGGTTAGC,

WT 3' arm reverse: GGTGAAATGCTTGACTCCTAGACTT,

WT 3' arm forward：CGTGGTGGAGCCGTTCTGTGAGACA.

PCR reactions were carried out under the following conditions:

WT 5’: 94°C for 2 min, followed by 30 cycles of 98°C for 10 s and 68°C for 4 min.

WT 3’: 94°C for 2 min; followed by 25 cycles of 98°C for 10 s, 61°C for 30 s, and 68°C for 2 min.

Target 5’: 94°C for 2 min, followed by 32 cycles of 98°C for 10 s and 68°C for 6 min.

Quantitative PCR for copy number confirmation – Copy number of the inserted cassette and random integration were confirmed by quantitative PCR. The puromycin resistance gene was quantified and normalized to the level of TATA box-binding protein (TBP) using SYBR Premix Ex Taq GC (Takara RR071A) and the Prism 7300 Real-time PCR system (ABI). Primer sequences were as follows: TBP (forward) CCCCCTCTGCACTGAAATCA and (reverse) GTAGCAGCACAGAGCAAGCAA; puromycin resistance (forward) CTCGACATCGGCAAGGTGTG and (reverse) GGCCTTCCATCTGTTGCTGC. The total volume of the reaction mixture was 30 μL, and primer concentrations were 0.5 μM each TBP primer and 0.2 μM each puromycin resistance gene primer. PCR was carried out under conditions of 95°C for 60 s; followed by 45 cycles of 95°C for 10 s and 60°C for 50 s; followed by 95°C for 15 s, 64°C for 60 s, and 99°C for 15 s. Absolute levels of PCR products were quantified using a standard curve. Because amplification products of the puromycin resistance gene often form dimers and outliers, samples were assayed in triplicate and outliers were omitted from the analysis. We analysed at least two lines of targeted clones that had only a single cassette inserted in the

Rosa26 locus.

ES cell differentiation system to detect precise circadian period length – A stock solution of 0.133 M RA was prepared by dissolving 50 mg RA (Sigma R2625) in 1.25 mL sterile DMSO (Nakalai). Aliquots were placed in amber 1.5-mL microtubes (Camlab), sealed with Parafilm, and stored at -80°C in the dark. To prepare a working solution for each experiment, DMSO was added to one aliquot to a final concentration of 0.01 M RA and solution was stored at -20°C for up to 3 weeks. Fresh working solution was used for every medium change and was handled with the fluorescent light in the laminar flow hood turned off. Cells were cultured from day 0 to day 8 in differentiation medium: Dulbecco's Modified Eagle Medium (DMEM, high glucose, pyruvate, Gibco 11995-065) containing 20% FBS, 0.1 mM MEM Non-Essential Amino Acids (Gibco), 100 U/mL penicillin and 100 µg/mL streptomycin (Gibco 15140-122), 2 mM L-glutamine (Gibco 25030-081), 100 µM β-mercaptoethanol (β-ME), and 1 µM RA. Fresh β-ME was prepared in DPBS without calcium and magnesium at the start of each experiment and filtered through a Millex GP 0.22-µm filter (Millipore) before addition to medium. RA was added to medium immediately before medium was added to cells. On day 0, ES cells were suspended in ES medium 2i(-)LIF(-) at a concentration of 1×10^6 cells/mL and plated in 10 mL differentiation medium on 0.2% gelatine-coated 100-mm dishes (Falcon 353003 or Iwaki 3020-100) and cultured at 37°C in a humidified atmosphere of 5% CO_2. The appropriate number of cells to plate so that the cultures are subconfluent on day 5 varies among cell lines and ranges from $3–9\times10^5$ cells/100-mm dish. Differentiation medium was prepared every 4 days, and medium was changed every other day after equilibration to 5% CO_2. On day 5, cells were washed with DPBS without calcium and magnesium, incubated with 1.5 mL

0.05% trypsin/0.48 mM EDTA (Gibco) at 37°C for 2 min, and pipetted up and down 10 times in 3 mL FBS using a 5-mL pipet. Cells were then sequentially applied to a 100-μm Cell Strainer (BD Falcon 352360) and 35-μm Cell Strainer (BD Falcon 352235). DMEM (7 mL) containing 10% FBS and 1× penicillin/streptomycin (Gibco) was added, followed by centrifugation at 1000 rpm (175–206×*g*) for 5 min. Supernatant was removed and cells were resuspended by pipetting five times with a blue tip. Cells were plated in 2 mL differentiation medium on 0.2% gelatine-coated 35-mm dishes (Falcon). The number of cells plated on day 5 is also finely adjusted so that cultures will be subconfluent on day 20, although it is similar among cell lines ($3–10\times10^5$/35-mm dish). On day 8, medium was changed to detection medium (DMEM without phenol red, without pyruvate, with 25 mM HEPES; Gibco) supplemented with 10% FBS, 1× penicillin/streptomycin (Gibco), and 1 μM RA. Medium was first equilibrated in CO_2; medium was changed every other day. We chose the strategy of pre-equilibration in detection medium because luminescence baselines are stabilized under these conditions.[15] On day 20, medium was changed to detection medium containing 1 μM RA, 10 μM forskolin, and 100 μM luciferin. Plates were sealed with silicone grease (Toray), and circadian oscillation was measured in a low-level light-detection unit (Hamamatsu photonics, C8801-01R) at 30°C in air.

Autocorrelation analysis of circadian period was carried out using Mathematica 9.0 (Wolfram) as previously described, with slight modification.[16] Briefly, bioluminescence time-series data were detrended by subtracting the trend curve of a 42-h timescale, calculated by the smoothing spline method with corresponding stiffness, and then used in the following analysis. Autocorrelation of the data was calculated within the range 15–30 h to determine the circadian period of oscillation, which was defined to provide the strongest autocorrelation. Statistical significance (with P= 0 as

most significant and P= 1 as least significant) of the circadian oscillation was evaluated by comparing the strongest autocorrelation with that of white noise ($P<0.01$). In the case of the C8801-01R light-detection unit, bioluminescence time series are produced every minute; we calculated the mean of measurements taken over 30 min and used these as time-series data with the circadian oscillation analysis tool.

Measurement of Per1 and Per2 half-life – NIH3T3 cells (ATCC) were maintained in DMEM, high glucose supplemented with 10% FBS (JRH 12603C-500ML Lot: 4C0702, 6K0357 or Sigma 17012-500ML Lot: S.12H183, heat inactivated), 100 U/mL penicillin and 100 μg/mL streptomycin (Gibco 15140-122). Cells were harvested every other day after incubation in 0.05% trypsin/0.48 mM EDTA (Gibco) and plated at a density of 2×10^5cells/100-mm dish (Falcon 353003 or Iwaki tissue culture dish 3020-100). For half-life measurement, cells were plated at 2×10^5 cells/35-mm dish on day 0. On day 1, cells were transfected with 1 μg pMU2- Per1::Luc or pMU2-Per2::Luc using FuGene6 (Promega) according to the manufacturer's protocol with Opti-MEM Reduced Serum Medium GlutaMAXSup (Gibco 51985-034) and UltraPure Water (Gibco) (Fugene 6 : DNA amount =3:1). On day 2, DMEM supplemented with 10% FBS, 100 U/mL penicillin, and 100 μg/mL streptomycin was added to each dish. On day 4, medium was changed to DMEM, high glucose (HEPES, no Phenol Red, Gibco 21063-029) supplemented with 10% FBS, 100 U/mL penicillin, 100 μg/mL streptomycin, 100 μM luciferin (Promega E1601), 400 μg/mL cycloheximide (Wako; 100 mg/mL stock solution prepared in DMSO and filtered through Millex GP 0.22-μm filter). Plates were sealed with silicone grease (Toray) and luminescence was measured with a low-level light-detection unit (Hamamatsu photonics, C8801-01R) and

Lm300122 software) at 30°C in room air. Exponential fitting to calculate half-life was performed with R (R Development Core Team) and the following code:

```
degradation <-read.csv("degradation.csv")
df <- data.frame(v=degradation$CH1_cpm, t=degradation$min)
out <- nls(v~a*exp(-k*t)+(100-a), data=df, start=list(a=100, k=0.001))
a=coef(out)[1]
k=coef(out)[2]
t_half_CH1=-(1/k)*log((a-50)/a)
t_half_CH1
rm(df,out,a,k)
```

CH1_cpm: counts per min (measurement data), min: minute, degradation: the name of csv file. In this code, protein half-life is produced with t_half_CH1.

Unfortunately, we could only perform 10 experiments at a time because only 10 low-level light-detection units were available. Thus, we repeated these experiments six times and were able to reproduce the results each time.

We tested several different types of plasmid DNA in these six independent experiments and used two different *Per1* cDNAs. We also tested both types of plasmids in *Per1* and *Per2*, one generated by restriction enzyme digestion and ligation and the other by infusion cloning (Takara). Moreover, we tested at least two plasmid DNA clones derived from different *E. coli* clones. We also confirmed that these results could be reproduced using DNA purified with the PureYield™ Plasmid Midiprep and Miniprep Systems (Promega). We also tested reversed-order experiments to refute that the difference in half-life originates from the experimental order. We

analysed all of the experiments in combination because consistent results were obtained. However, inter-experimental variance was substantial, most likely because of the phase difference between the experiments, the time lag between the media change and the start of the measurements (the biological safety cabinet and the low temperature incubator were located in different rooms), and resetting effects following the media change. Hence, we likewise performed these experiments using other conditions in which we dispersed the cell phases.

In the case of the dispersed phase conditions, the procedure was generally the same as the procedure detailed above, with the exceptions that CHX was not added prior to starting the measurements, we waited six days after the media change to open or touch the incubator, the temperature was not changed, and care was taken not to vibrate the dishes. Six days later (Day 10), we gently removed the dishes from the incubator and carefully placed them on a table directly in front of the incubator in a room that was slightly warmed (25–30°C). Next, we immediately opened the sealed dishes, dispensed 8 μL of CHX (400 mg/mL) homogeneously onto the surface of the media using a 200 μL tip, and quickly but gently rocked the dishes twice, taking care not to agitate the cells and not to expose the cells to strong light. Afterward, we quickly placed the cells back in the low-level light-detection units, gently closed the incubator, and started the measurements. The important point of this operation was not to expose the cells to a physical stimulus such as swirling, mixing, or a temperature change, which could affect the phase of the cells. We also had concerns regarding light absorption of the luciferin-containing media, which could cause changes in the baseline measurements and make it difficult to accurately calculate the half-life.

Sample preparation for Western Blotting

ES cells: Subconfluent Per2-rescued (#1) and Per1-rescued (#16) *Per2*$^{(-/-)}$ ES cells (2 days after spreading 1×10^6 cells in a 35-mm dish), were washed twice with cold DPBS without calcium and magnesium. Next, 216 μL of RIPA buffer with cOmplete™ Mini Protease Inhibitor cocktail (1X) and PhosSTOP™ (1X) was added, followed by pipetting 30 times using a 1000 μL tip and collecting the lysates into 1.5 mL tubes. The tubes were then rotated at 4°C for 1 h and stored at -80°C. The final protein concentration was 2.7–3.4 μg/μL (4 dishes).

Control NIH3T3 cells: NIH 3T3 cells were plated at a density of 2×10^5 cells/35-mm dish on day 0. On day 1, the cells were transfected with 1 μg pMU2, pMU2-Per1, or pMU2-Per2 using FuGENE® 6 transfection reagent (Promega) according to the manufacturer's protocol with Opti-MEM® Reduced Serum Medium, GlutaMAX™ Supplement (Gibco 51985-034) and UltraPure Water (Gibco) [FuGENE® 6 (μL): DNA amount (μg) =3:1]. On day 2, we added 1 mL of media to each dish. On day 4, we lysed the cells with 216 μL of RIPA buffer with cOmplete™ Mini Protease Inhibitor cocktail (1X) and PhosSTOP™ (1X) and mixed by pipetting 20 times using a 1000 μL tip and collection of the lysates into 1.5 mL tubes. The tubes were then rotated at 4°C for 1 h and stored at -80°C. The final protein concentration was within the range of 1.07–1.13 μg/μL (6 dishes).

Half-life analysis: NIH 3T3 cells were plated at a density of 1.2×10^6 cells/100-mm dish on day 0. On day 1, the cells were transfected with 6 μg pMU2-Per1 or pMU2-Per2 using FuGENE® 6 transfection reagent (Promega) according to the manufacturer's protocol with Opti-MEM® Reduced Serum Medium, GlutaMAX™ Supplement (Gibco 51985-034) and UltraPure Water (Gibco). On day 2, the cells in each of the 100-mm dishes were divided into six dishes, each at a

final density of approximately 2.4×10^5 cells/35-mm dish. On day 4, the medium was changed to 2 mL of DMEM, high glucose (HEPES, no Phenol Red, Gibco 21063-029) supplemented with 10% FBS, 100 U/mL penicillin, and 100 μg/mL streptomycin. The dishes were sealed with silicone grease (Toray) and incubated for 4 days at 30°C in room air using a low temperature incubator (EYELA, LTI 700E).

Prior to the addition of CHX on Day 8, we warmed the room slightly (greater than 25°C) to prevent the dishes from becoming cold. CHX was not added to the NT (no treatment) control dishes, and we lysed the cells prior to the addition of CHX to the other dishes. CHX was added to the time 0 h sample and the cells were immediately lysed.

On day 8, we gently removed the dishes from the incubator and carefully placed them on a table in front of the incubator. We then immediately opened the sealed dishes, dispensed 8 μL of CHX (400 mg/mL) homogeneously onto the surface of the media using a 200 μL tip, and quickly but gently rocked the dishes twice, taking care not to agitate the cells. The plates were then quickly placed back inside the incubator. The important point of this operation was not to expose the cells to a physical stimulus such as swirling, mixing, or a temperature change, which could affect the phase of the cells.

At 0, 1, 2, 4, and 8 h after the CHX treatment, we lysed the cells with 216 μL of RIPA buffer with cOmplete™ Mini Protease Inhibitor cocktail (1X) and PhosSTOP™ (1X), and mixed by pipetting 35 times with a 1000 μL tip. The final protein concentration was within the range of 1.30–1.55 μg/mL.

Western Blotting - Western blotting was performed as previously described[17-19] with slight

modifications. The cell culture dish was washed twice with ice-cold DPBS without calcium and magnesium and the cells were homogenized by pipetting in RIPA buffer [50 mM Tris-HCl (pH 7.5), 150 mM NaCl, 1% NP40, 0.1% SDS, and 0.5% sodium deoxycholate] containing protease inhibitor (Roche, cOmplete™ Mini Protease Inhibitor cocktail, 11836153001, 1 tablet for 10 mL) and phosphatase inhibitor (Roche, PhosSTOP™ Phosphatase Inhibitor Cocktail, 04906837001, 1 tablet for 10 mL) using a blue tip. The samples were rotated vertically on a rotary shaker (Iwaki, RCC-100, rotating speed： 1) for 1 h soon after lysis followed by storage at -80°C. After thawing on ice, the samples were centrifuged at 12,000 rpm for 20 min at 4°C. The supernatants were collected and the total protein concentrations were quantified using the Pierce BCA Protein Assay Kit (Thermo Scientific, 23227), the iMark™ Microplate absorbance reader (Bio-Rad), and a 96-well flat bottom plate (Iwaki) with BSA as the standard. The protein concentrations were equalized then denatured by boiling at 95°C for 5 min in NuPAGE® LDS Sample Buffer (Thermo, NP0007) and NuPAGE® Sample Reducing Agent (Thermo, NP0009). Each sample containing equal amounts of protein (12, 15, or 15.75 μg) was subjected to sodium dodecyl sulfate-polyacrylamide gel electrophoresis (SDS-PAGE) using SuperSep™ Ace 7.5% 13 well gels (Wako, 198-14941). Electrophoresis was performed with a Double Mini Gel Electrophoresis Apparatus (Nippon EIDO) and a BioCRAFT Real Power (Model BP4) power source at 20 mA for approximately 90 min. Precision Plus Protein™ Dual Colour Standards (Bio-Rad, 161-0374) were used as the molecular weight marker. The proteins were then transferred to a polyvinylidene fluoride (PVDF) membrane (Pall Life Science, FluoroTrans ® W 0.2 µm membrane, EH2222) for 1 h at 80 mA using TE 70 PWR semi-dry Transfer Unit (GE), filter paper (Whatman® 3030-928) and film (BioCRAFT C9032). After blocking for 1 h in 5% skim milk (Yukijirushi) at room temperature with shaking

at 60 rpm (TAITEC, NR-2), the primary antibodies [anti- mouse PER2 (Rabbit; Alpha Diagnostic International, PER21-A), anti-Per1 (Mouse) pAb (Guinea pig; MBL, PM091), monoclonal anti-FLAG M2 antibody produced in mouse (Sigma F1804-50UG), and monoclonal anti-β-actin antibody produced in the mouse clone AC-15, ascites fluid (Sigma A5441)] were diluted 1000× in Tris-buffered saline solution/0.1%Tween (TBST) containing 5% skim milk, added to the membranes, and the membranes and primary antibodies were incubated at 4°C. The lone exception was β-actin, which was diluted 5000×. The membranes and antibody solutions were contained in a PP laminate plastic bag (BM Bio, PS-I-100) that was sealed using a polysealer (Fuji Impulse, P-200). The four corners of the sealed bags were adhered to the rotary shaker disc (Iwaki, RCC-100) with packaging tape (Rinrei) and rotated vertically. After incubation, the membranes were washed three times for 10 min each in TBST and incubated with the secondary antibody in 5% skim milk for 1 h at room temperature using a rotary shaker (TAITEC, NR-2) at 60 rpm. Secondary antibodies [Rabbit anti-guinea pig IgG (H+L) secondary antibody (HRP conjugate; Invitrogen 61-4620), ECL™ anti-mouse IgG, horseradish peroxidase-linked species-specific whole antibody (from sheep; GE, NA931), anti-rabbit IgG, HRP-linked whole Ab (from donkey; GE, NA934V)] were diluted 2000×, except for anti-mouse HRP antibody, which was diluted 5000× with anti β-actin as the primary antibody. After six washes in TBST for 10 min each, the membranes were washed with Milli-Q® water, drained using KimWipes™ (Kimberly Clark), then ECL™ Prime Western Blotting Detection Reagent (GE, RPN2232) was added to the membranes, followed by mixing several times and incubation in the PP laminate plastic bag (BM Bio, PS-100) for at least 1 min. The membranes were then drained using KimWipes™ and held in another PP laminate Plastic bag (BM Bio, PS-100), followed by exposure to Hyperfilm ECL (GE, 28906837) in the film cassette

(Okamoto, PL-Btype 8×10 inch). The film was developed using a TCX-101 developing machine (Konica Minolta). The films were scanned using a GT-X750 scanner (Epson) in the positive film mode. Reblotting was performed only for β-actin. The β-actin membranes were washed three times with TBST for 5 min each, then treated with WB Stripping Solution (Nakalai, 05364-55) for 30 min. Afterward, the membranes were washed three times for 5 min in TBST and blocked again.

Other statistical analysis – Comparisons of the mean circadian period, periodicity, and half-life of two groups of samples were carried out using Student's *t*-test (bilateral, unequal variances). Error bars represent standard deviation (SD). From a statistical viewpoint, *t*-tests can be performed and a P-value can be calculated using only two samples,[20] although the reliability may be low. For the comparison of three or more groups, we applied ANOVA and Tukey's post hoc tests using SPSS 26 (IBM).

Supplemental References

1 Yamajuku, D. *et al.* Identification of functional clock-controlled elements involved in differential timing of Per1 and Per2 transcription. *Nucleic Acids Res* **38**, 7964-7973, doi:10.1093/nar/gkq678 (2010).

2 Albrecht, U., Sun, Z. S., Eichele, G. & Lee, C. C. A differential response of two putative mammalian circadian regulators, mper1 and mper2, to light. *Cell* **91**, 1055-1064 (1997).

3 Ukai, H. *et al.* Melanopsin-dependent photo-perturbation reveals desynchronization underlying the singularity of mammalian circadian clocks. *Nat Cell Biol* **9**, 1327-1334, doi:10.1038/ncb1653 (2007).

4 Minami, Y. *et al.* Measurement of internal body time by blood metabolomics. *Proceedings of the National Academy of Sciences of the United States of America* **106**, 9890-9895, doi:10.1073/pnas.0900617106 (2009).

5 Richter, C. P. *A behavioristic study of the activity of the rat.* (Williams & Wilkins Company, 1922).

6 Sokolove, P. G. & Bushell, W. N. Chi Square Periodogram - Its Utility for

Analysis of Circadian-Rhythms. *J Theor Biol* **72**, 131-160, doi:Doi 10.1016/0022-5193(78)90022-X (1978).
7 Bae, K. *et al.* Differential functions of mPer1, mPer2, and mPer3 in the SCN circadian clock. *Neuron* **30**, 525-536 (2001).
8 Refinetti, R. Laboratory Instrumentation and Computing - Comparison of 6 Methods for the Determination of the Period of Circadian-Rhythms. *Physiol Behav* **54**, 869-875, doi:Doi 10.1016/0031-9384(93)90294-P (1993).
9 Usui, S., Takahashi, Y. & Okazaki, T. Range of entrainment of rat circadian rhythms to sinusoidal light-intensity cycles. *Am J Physiol Regul Integr Comp Physiol* **278**, R1148-1156 (2000).
10 Yoo, S. H. *et al.* PERIOD2::LUCIFERASE real-time reporting of circadian dynamics reveals persistent circadian oscillations in mouse peripheral tissues. *Proceedings of the National Academy of Sciences of the United States of America* **101**, 5339-5346, doi:10.1073/pnas.0308709101 (2004).
11 Kiyonari, H., Kaneko, M., Abe, S. & Aizawa, S. Three inhibitors of FGF receptor, ERK, and GSK3 establishes germline-competent embryonic stem cells of C57BL/6N mouse strain with high efficiency and stability. *Genesis* **48**, 317-327, doi:10.1002/dvg.20614 (2010).
12 Wataya, T. *et al.* Minimization of exogenous signals in ES cell culture induces rostral hypothalamic differentiation. *Proceedings of the National Academy of Sciences of the United States of America* **105**, 11796-11801, doi:10.1073/pnas.0803078105 (2008).
13 Ying, Q. L. *et al.* The ground state of embryonic stem cell self-renewal. *Nature* **453**, 519-523, doi:10.1038/nature06968 (2008).
14 Niwa, H., Ogawa, K., Shimosato, D. & Adachi, K. A parallel circuit of LIF signalling pathways maintains pluripotency of mouse ES cells. *Nature* **460**, 118-122, doi:10.1038/nature08113 (2009).
15 Isojima, Y. *et al.* CKIepsilon/delta-dependent phosphorylation is a temperature-insensitive, period-determining process in the mammalian circadian clock. *Proceedings of the National Academy of Sciences of the United States of America* **106**, 15744-15749, doi:10.1073/pnas.0908733106 (2009).
16 Ukai-Tadenuma, M., Kasukawa, T. & Ueda, H. R. Proof-by-synthesis of the transcriptional logic of mammalian circadian clocks. *Nat Cell Biol* **10**, 1154-1163, doi:10.1038/ncb1775 (2008).
17 Akiyoshi, T. *et al.* A novel organ culture model of aorta for vascular calcification. *Atherosclerosis* **244**, 51-58, doi:10.1016/j.atherosclerosis.2015.11.005 (2016).
18 Tamiya, H. *et al.* Analysis of the Runx2 promoter in osseous and non-osseous cells and identification of HIF2A as a potent transcription activator. *Gene* **416**, 53-60, doi:10.1016/j.gene.2008.03.003 (2008).
19 Ode, K. L., Fujimoto, K., Kubota, Y. & Takisawa, H. Inter-origin cooperativity of geminin action establishes an all-or-none switch for replication origin licensing. *Genes to cells : devoted to molecular & cellular mechanisms* **16**, 380-396, doi:10.1111/j.1365-2443.2011.01501.x (2011).
20 de Winter, J. C. F. Using the Student's t-test with extremely small sample sizes.

Practical Assessment,Research & Evaluation **18**, 1-12 (2013).

Tamiya_FigS1

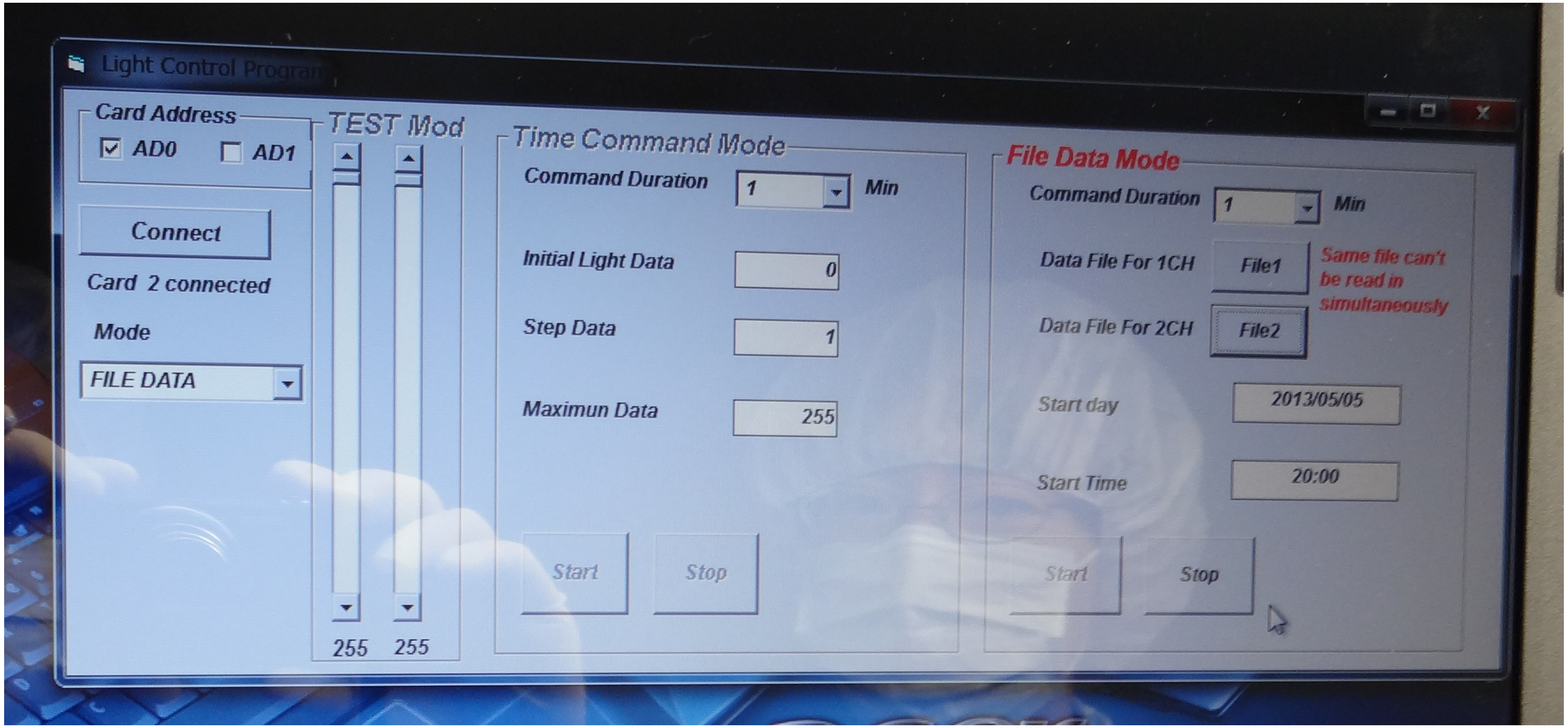

Tamiya_FigS2

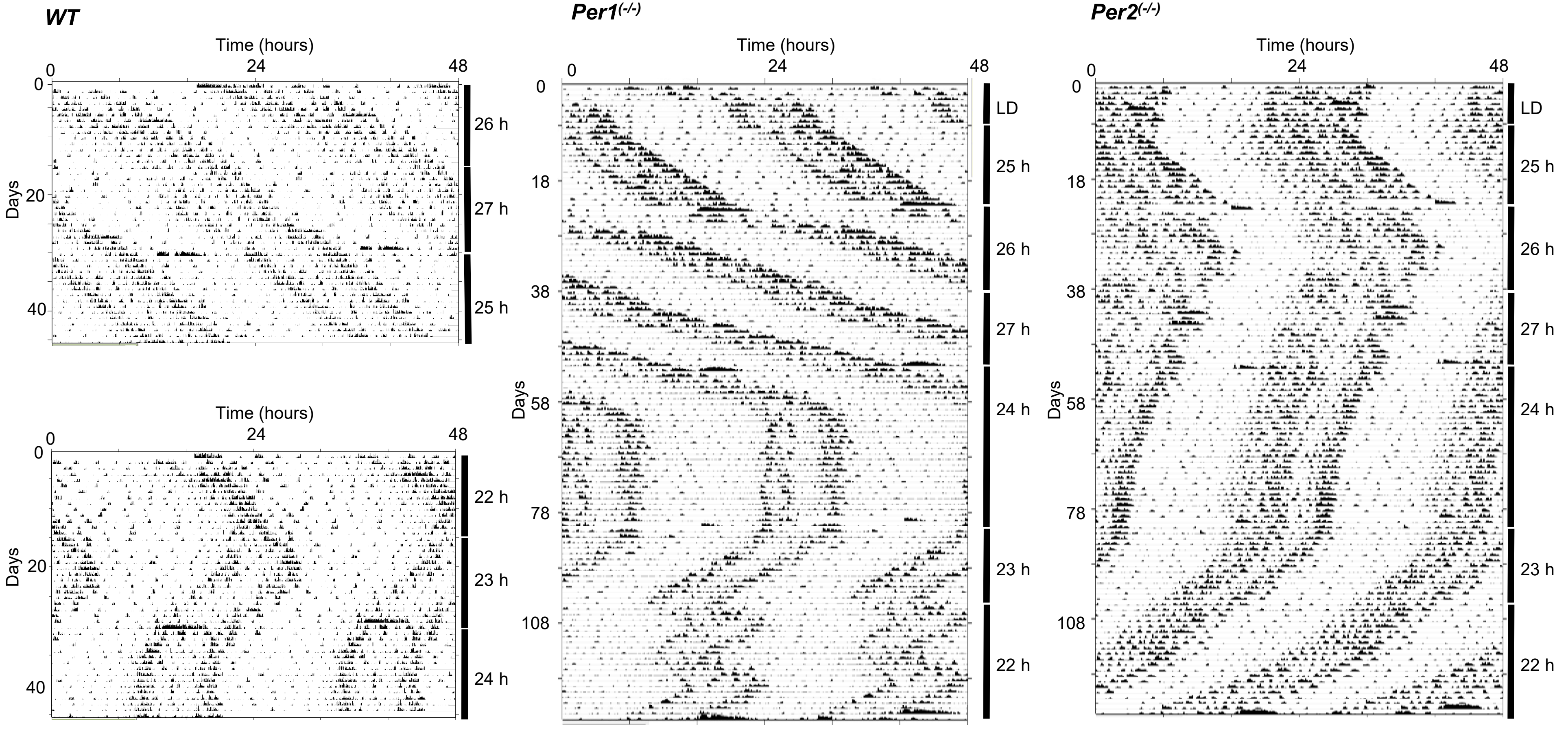

Tamiya_FigS3

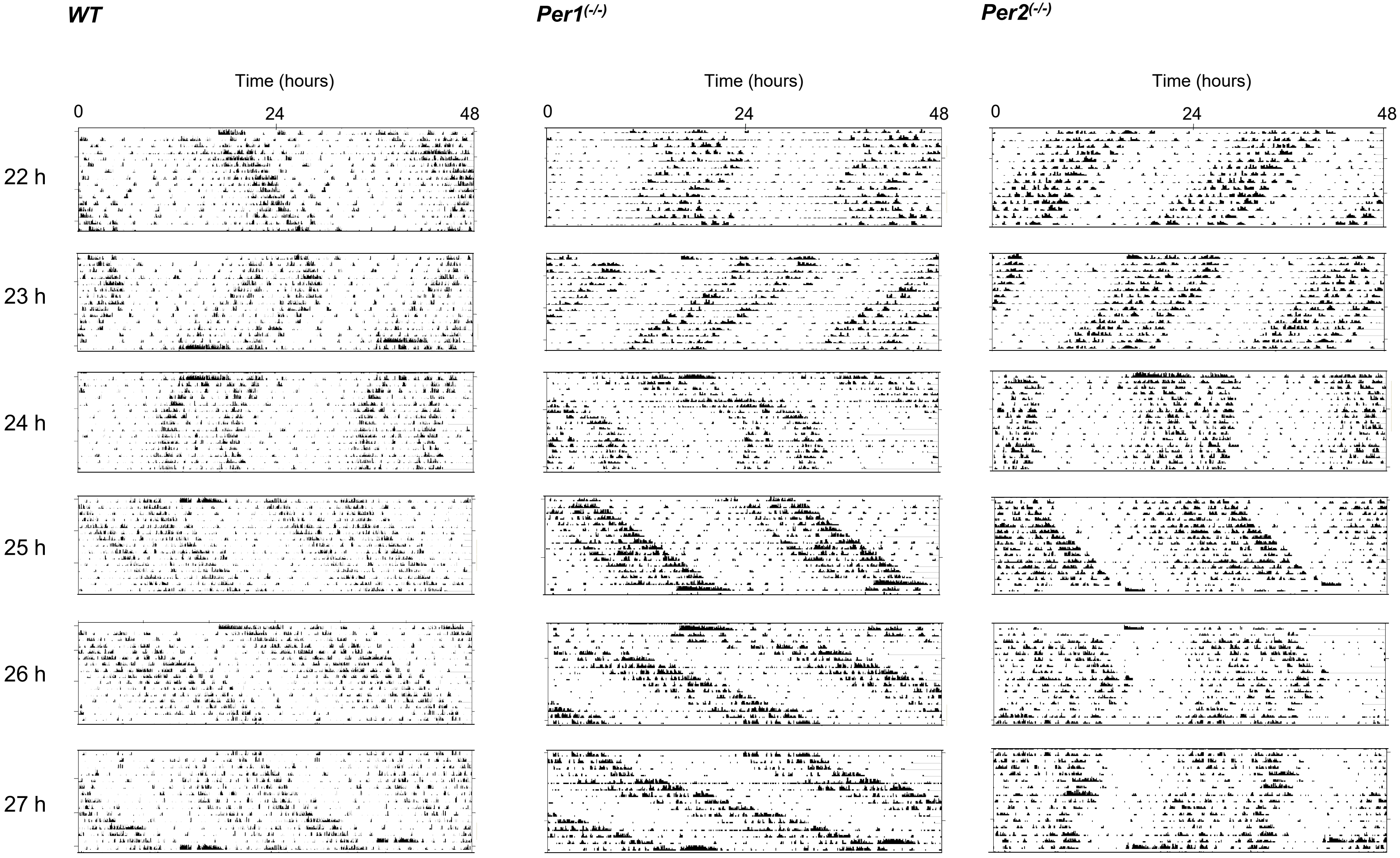

Tamiya_FigS4

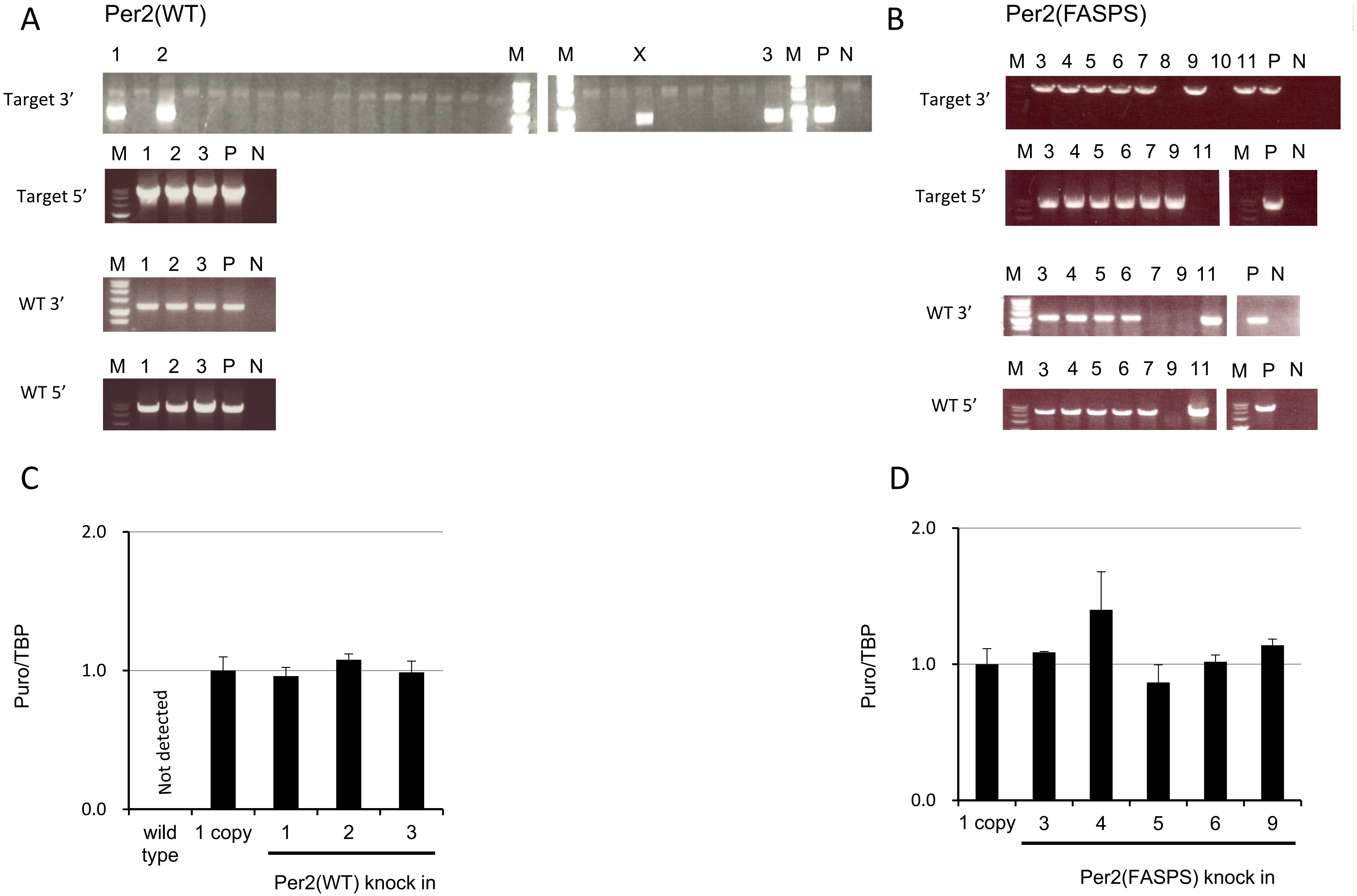

Tamiya_FigS5

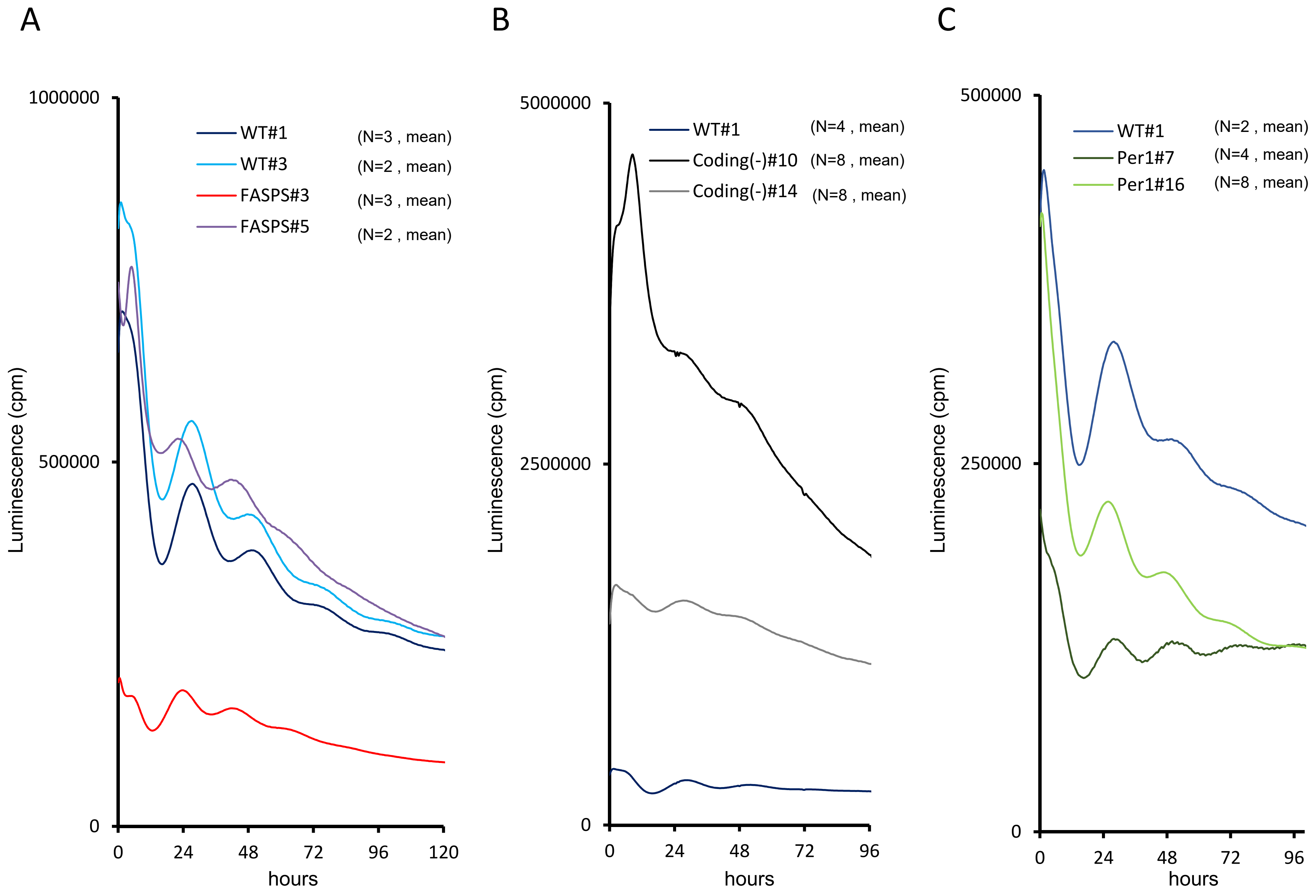

Tamiya_FigS6

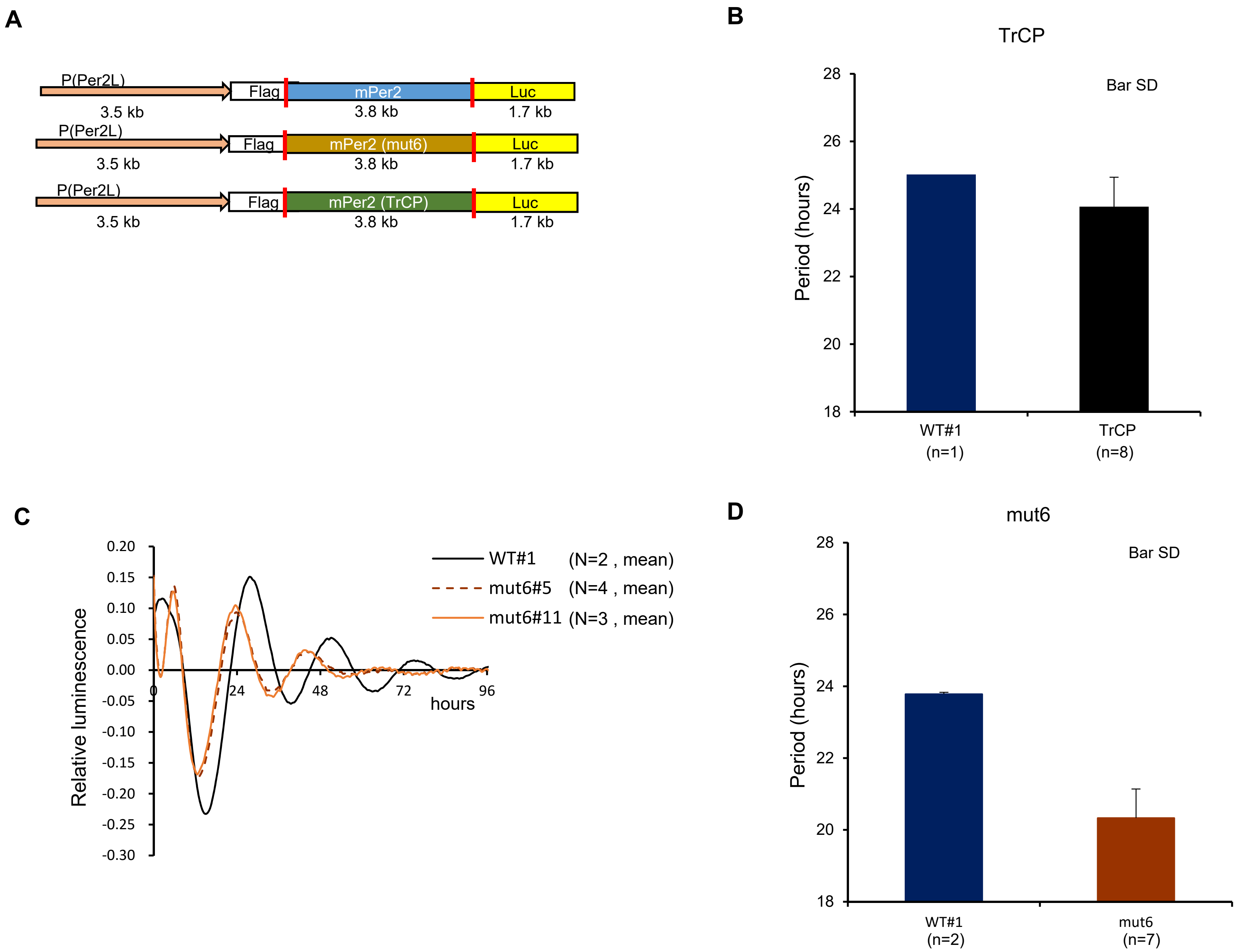

Tamiya_FigS7

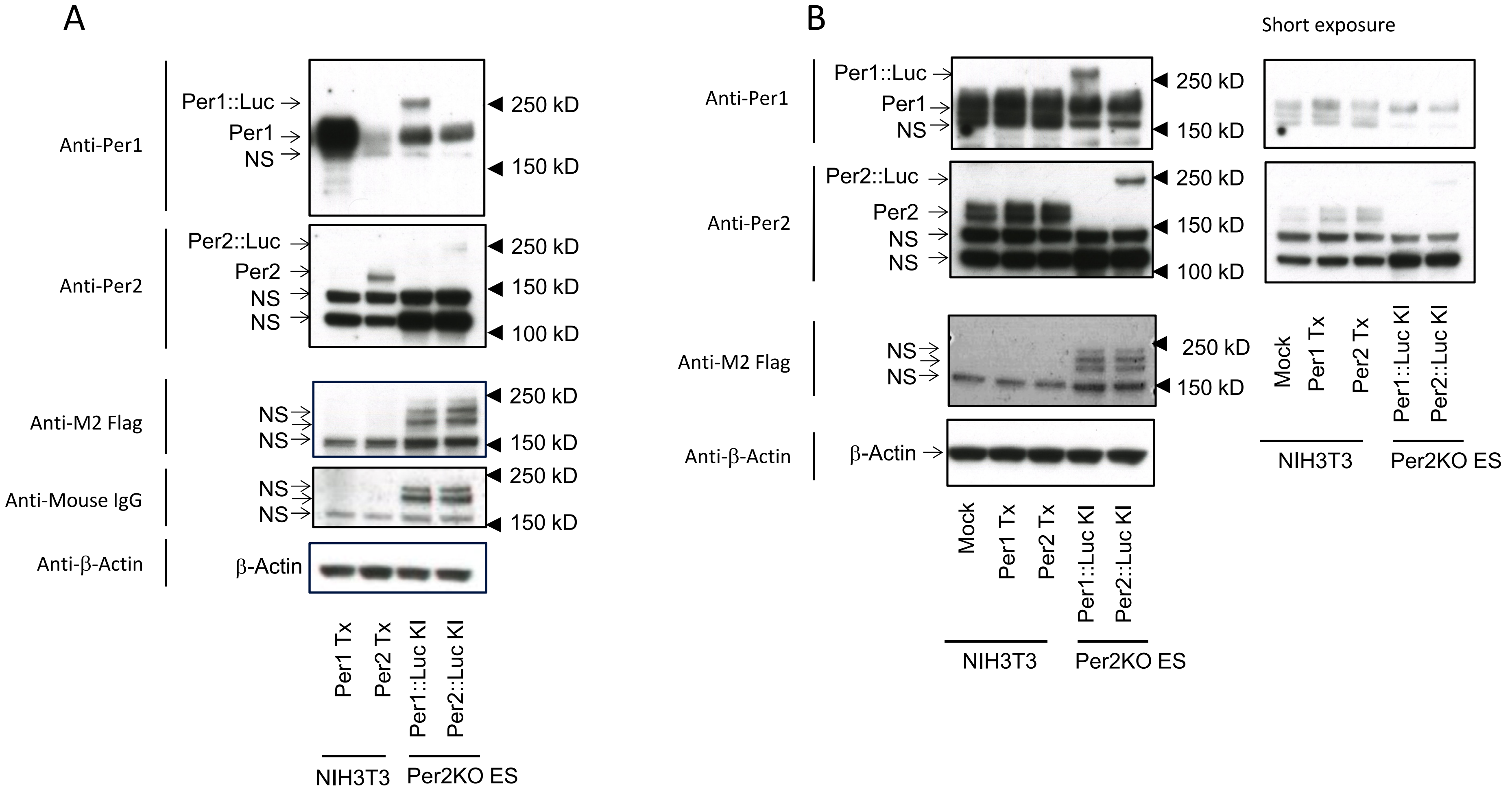

Tamiya_FigS8

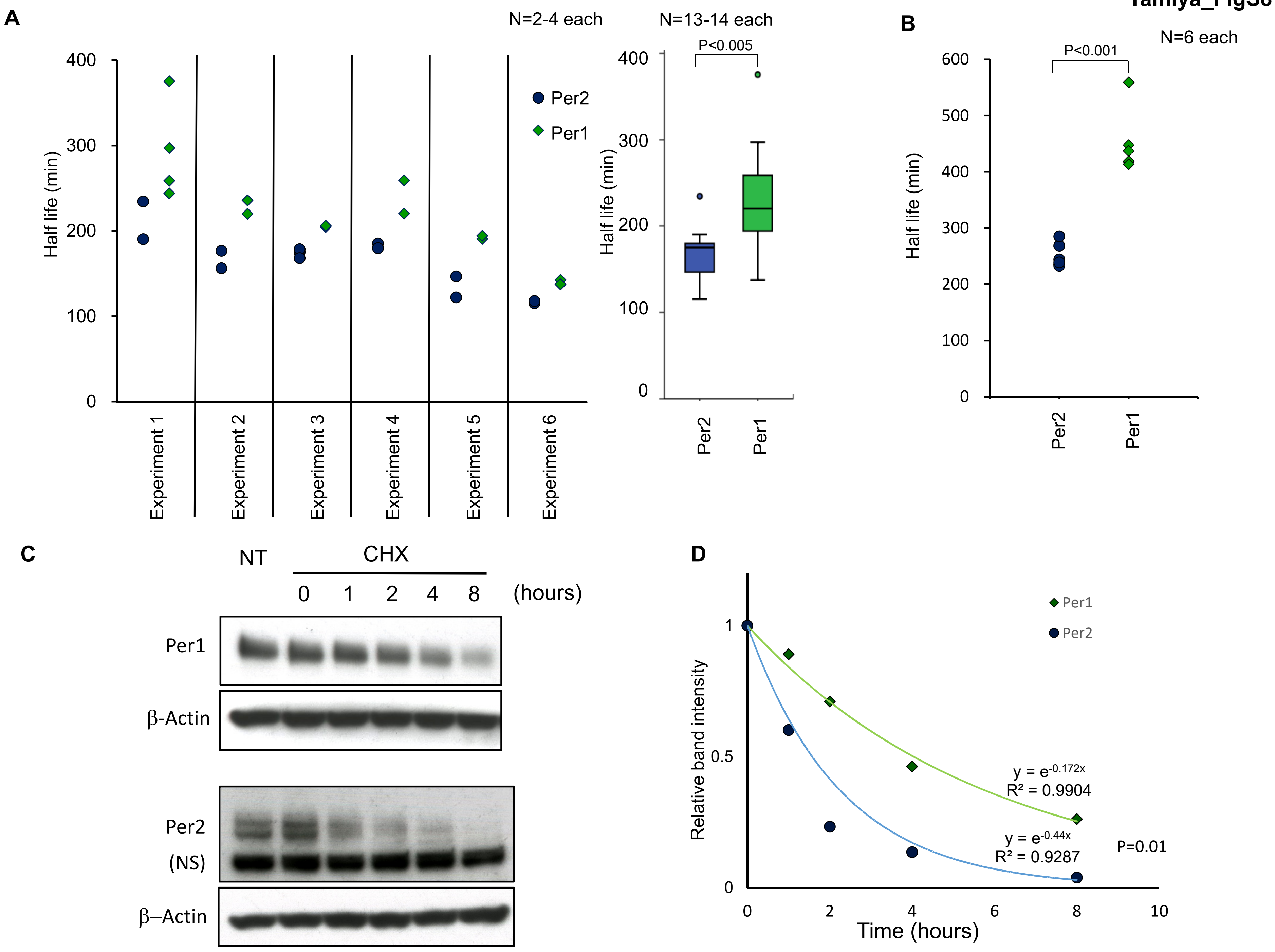

A

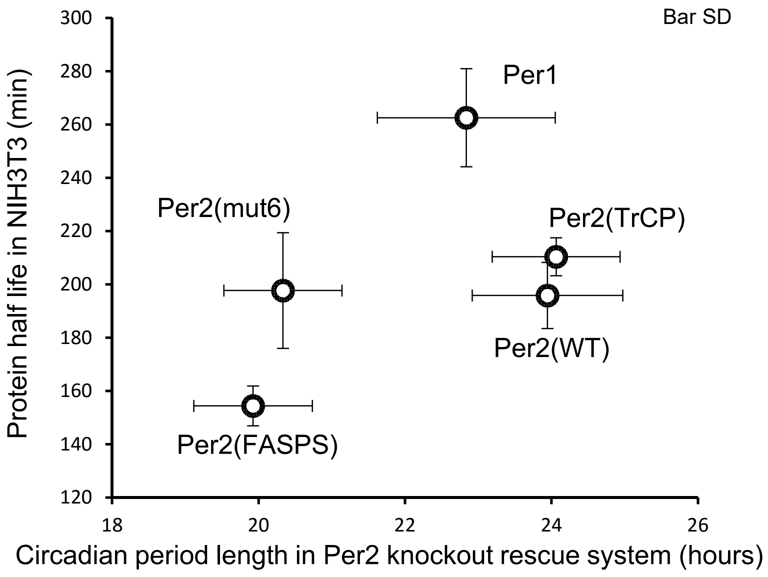
Bar SD
Per1
Per2(mut6)
Per2(TrCP)
Per2(WT)
Per2(FASPS)
Protein half life in NIH3T3 (min)
300
280
260
240
220
200
180
160
140
120
18
20
22
24
26
Circadian period length in Per2 knockout rescue system (hours)

B

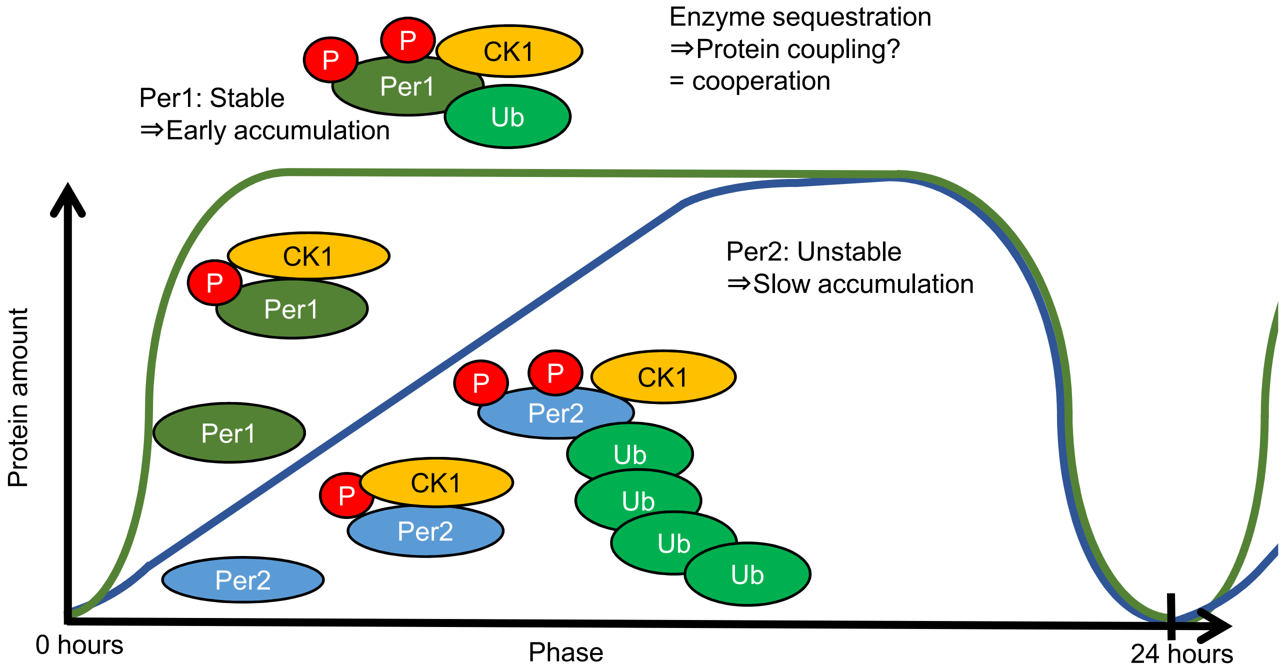
Enzyme sequestration
⇒Protein coupling?
= cooperation
Per1: Stable
⇒Early accumulation
Per2: Unstable
⇒Slow accumulation
P
CK1
Per1
Ub
Per2
Protein amount
0 hours
Phase
24 hours
